# Clinical and immunological effects of mRNA vaccines in malignant diseases

**DOI:** 10.1186/s12943-021-01339-1

**Published:** 2021-03-15

**Authors:** Annkristin Heine, Stefan Juranek, Peter Brossart

**Affiliations:** Medical Clinic III for Oncology, Hematology, Immune-Oncology and Rheumatology, University Hospital Bonn, Venusberg Campus 1, 53127 Bonn, Germany

**Keywords:** mRNA vaccine, Cancer, COVID19, Immune response, Tumor-associated antigen, T cells, B cells

## Abstract

*In vitro*-transcribed messenger RNA-based therapeutics represent a relatively novel and highly efficient class of drugs. Several recently published studies emphasize the potential efficacy of mRNA vaccines in treating different types of malignant and infectious diseases where conventional vaccine strategies and platforms fail to elicit protective immune responses. mRNA vaccines have lately raised high interest as potent vaccines against SARS-CoV2. Direct application of mRNA or its electroporation into dendritic cells was shown to induce polyclonal CD4+ and CD8+ mediated antigen-specific T cell responses as well as the production of protective antibodies with the ability to eliminate transformed or infected cells. More importantly, the vaccine composition may include two or more mRNAs coding for different proteins or long peptides. This enables the induction of polyclonal immune responses against a broad variety of epitopes within the encoded antigens that are presented on various MHC complexes, thus avoiding the restriction to a certain HLA molecule or possible immune escape due to antigen-loss. The development and design of mRNA therapies was recently boosted by several critical innovations including the development of technologies for the production and delivery of high quality and stable mRNA. Several technical obstacles such as stability, delivery and immunogenicity were addressed in the past and gradually solved in the recent years.

This review will summarize the most recent technological developments and application of mRNA vaccines in clinical trials and discusses the results, challenges and future directions with a special focus on the induced innate and adaptive immune responses.

## Introduction

Although remarkable progress has been achieved during the last decade to combat cancer, it is still the world’s second most leading cause of death [1]. Besides surgery, radiation and chemotherapy as essential columns of anti-tumor treatment, immunotherapy and targeted therapies have lately revolutionized and complemented anti-tumor therapy [2, 3]. The tremendous success of checkpoint inhibitors (CPI) for a broad variety of malignant diseases has generated new interest in immunotherapeutic approaches to fight cancer [2]. Amongst those, tumor-specific vaccines have had a long history of intensive research and clinical application, however with limited success in the past. The optimal route and vehicle of application, the choice of the appropriate adjuvant and the correct identification of the target antigen have been shown to be of crucial importance [4]. The basis of all these developments is the fact that T cells can eliminate cancer cells. They have to recognize tumor-specific antigens and need to be generated in patients in sufficient amounts [5]. For the progress of T-cell associated cytotoxicity, the identification of tumor-specific antigens set a milestone and paved the way for its application as tumor vaccines [6].

Individualized messenger RNA (mRNA) vaccines are in some cases applied together with cytokines [7] or bacterial and viral adjuvants which trigger pattern recognition receptors (PRRs). mRNA vaccines were shown to generate potent and protective immune responses that consist of cellular and humoral components and are capable to eliminate malignant or infected cells [8, 9]. They can be delivered by direct injection of naked or stabilized RNA into lymph nodes, subcutaneous or intradermal application, electroporated into *ex vivo* generated antigen presenting cells (APC) and complexed with protamine or nanoparticles [8]. More importantly, compared with many of the current vaccination strategies (such as DNA vaccines), the production of mRNA is faster, more flexible and less expensive and it can be used for precise and individualized therapies [10]. mRNA vaccination further allows a rapid and safe production of vaccines in pandemics such as SARS-CoV2 [11]. In addition, mRNAs allow the development of personalized patient-specific vaccines based on sequencing results of tumor samples that can be rapidly adapted [8, 9, 12]. mRNA vaccines can be produced without the comprehensive and time-consuming manufacturing problems that are associated with the production of plasmid DNA, viral vectors or recombinant proteins. In contrast to plasmid DNA, mRNA is independent of active cell division and is effective in mitotic and non-mitotic cells. Unlike the viral vectors or plasmid DNA that mediate long term expression of the target genes, which can potentially trigger anti-DNA antibodies and autoimmunity, mRNA application results in a rapid and transient expression of the encoded protein or peptide with the duration of a few days or weeks that makes mRNA easier to control [8, 13]. Importantly, mRNAs will not be integrated into the host genome [8, 9] which is an essential safety issue.

### mRNA production and stabilization

There are two classes of mRNAs, non-replicating and self-amplifying, that are currently used. Non-replicating mRNA encodes only the target antigen, while self-amplifying mRNA vaccines also encode the replication machinery of a virus. This results in an increase not only in the duration and level of antigen expression, but also an enhanced vaccine-induced immune response. Self-amplifying mRNA and non-replicating mRNA vaccines are used for infectious diseases [14], while non-replicating mRNA is utilized in cancer vaccines [8, 9].

Engineered *in vitro* transcribed (IVT) RNA resembles the naturally processed and matured mRNA in the cytoplasm of eukaryotic cells. Upon vaccination and cellular uptake at the site of application the RNA is transported to the cytoplasm. There the cellular translation machinery synthesizes the encoded protein that subsequently undergoes post-translational modifications yielding a properly folded functional protein. This process is of particular interest for the transient expression of antigen-specific T cell receptors (TCR) [15] or chimeric antigen receptors (CARs) in peripheral blood lymphocytes which are used for adoptive T cell therapies [16].

IVT of mRNA is a well-established procedure [17, 18]. It can be routinely performed in a cell-free approach and yields high amounts of RNA. First, a DNA template harboring a primer-binding site for the utilized RNA polymerase (e.g. T7, T3 or SP6 phage RNA polymerase) [19] needs to be designed. This DNA template should as a minimum contain the open reading frame (ORF) of the protein of interest and flanking untranslated regions (5’ and 3’ UTR). Characteristic for fully processed mature mRNA is also the presence of a 5’ cap and a 3’ poly(A) tail [20].

The canonical 5’ cap structure in eukaryotic cells is an inverted 7-methylguanosine (m7G), which is added co-transcriptionally to the first nucleotide of the mRNA via a 5’-5’ triphosphate bridge. The function of the 5’ cap is to increase the stability and translational efficacy of the mRNA and also to remove its immunogenicity. IVT mRNAs exhibit a 5’ triphosphate moiety, which is highly immunogenic. Triphosphorylated mRNA are recognized in the cytoplasm by PRRs and cause a type-1 interferon (IFN1) response [21]. To prevent the recognition of the IVT mRNA as ‘foreign’, the triphosphate has to be removed and a 5’ cap needs to be added. There are several strategies to achieve this [22]. Capping can be done co-transcriptionally by adding a cap-analog to the IVT reaction. The addition of a cap analog harbors the risk that it will be incorporated in the wrong orientation, yielding the mRNA to be translation-incompetent. The development of anti-reverse cap analogs (ARCA) forces the polymerase to incorporate the ARCA in the correct forward orientation [23] . Capping can also be done post-transcriptional by removing the triphosphate with a phosphatase and adding a m7G by a 2’-O-methyltransferase. Both, co- and post-transcriptional capping bear the risk that not all mRNA molecules will be modified, which leads to increased immunogenicity [24], caused by the activation of PRRs by wrongly capped mRNAs.

The poly(A) tail can already be part of the DNA template but it can also be added post-transcriptionally using a poly(A) polymerase (PAP) [25]. The poly(A) tail should be 100-250 nucleotides long. The optimal length of the poly(A) tail depends on the target cell type. The poly(A) tail increases the stability of the mRNA and its translational efficacy. The use of modified adenosines can further increase the stability of the poly(A) tail against degradation by cellular RNases [26]. The addition of a poly(T) stretch to the DNA template is preferable, because it gives better control about the precise length of the tail and makes subsequent enzymatic manipulation of the mRNA obsolete.

The UTRs have important cellular functions and are responsible for the regulation of the translation of the mRNA and thus protein expression [27]. The 5’ UTR is in concert with the 5’ cap important during the initiation of the first round of translation and the formation of the pre-initiation complex. However, the addition of internal ribosome entry sites (IRES) makes the formation of the pre-initiation complex obsolete, because the ribosome is directly recruited to the mRNA [28]. Of note, the 5’ cap is still important for the stability of the mRNA. The 5’ UTR should also contain the Kozak consensus sequence, which also contributes to successful initiation of translation. The Kozak consensus sequence serves as an optimized protein translation initiation site in eukaryotes [29]. The 3’ UTR was shown to also increase translational efficacy by including certain sequences, such as sequences from the α- or β-globin [30]. Generally, a high GC- and low U-content are favorable to minimize immunogenicity and maximize the stability of the IVT mRNA [31].

The ORF itself encodes the protein of interest. It can also influence the translation. Several codon optimization strategies were developed that aim to optimize the translational process [32]. These strategies are based on the fact that most amino acids are encoded by several codons. Some codons are rare and less efficient during translation. However, manipulation of the original sequence can also lead to unwanted effects, because studies showed that even synonymous mutations could contribute to the pathogenesis of complex human diseases [33].

During IVT, modified nucleotides can be used to further stabilize the IVT mRNA and also lower the immunogenicity [34]. Common substitutions are adenosine with N6-methyladenosine (m6A), cytidine with 5-methylcytidine (m5C) or uridine with 5-methyluridine (m5U), 2-thiouridine (s2U) or pseudouridine (ψ) to name a few. Especially m5C and ψ were reported to reduce the immunogenicity and even increase the translation efficiency of IVT mRNA [34].

After IVT the mRNA needs to be purified and its concentration needs to be determined. Special care must be taken to eliminate aberrant, truncated and degraded products. Purification for a clinical application is done by performing chromatography-based techniques in order to gain a clean mRNA devoid of shorter fragments caused by abortive initiation or double-stranded (ds) RNA caused by self-complementary 3’ extension, both a common cause for impurities. Recently, an alternative method for the purification of mRNA was presented, based on adsorption to cellulose, in order to remove dsRNA from the transcribed mRNA [35]. To note, it has been demonstrated that successful mRNA translation and protein expression can be achieved without using any modified nucleotides and relies more on the purity of the mRNA and the sequence composition of its single parts [36].

### Strategies for improving mRNA delivery

Different ways of vaccine delivery have been intensively investigated for the efficient cellular uptake and biodistribution (reviewed in [8, 37]). First, dendritic cell (DCs) can internalize RNAs by endocytosis and this process can be improved by electroporation [38]. External loading of DCs facilitates efficient targeting of APCs, but requires adoptive cell transfer and is time and labor-intensive. Second, naked RNA can be injected with or without a carrier. Carriers help to improve stability, RNA uptake and translatability of vaccines. Several carriers have been developed [39]. Amongst those, lipid nanoparticles (LNPs) have evolved to become the most promising. These LNPs are non-viral carriers that are easy-to-produce and considered non-toxic. They usually contain cholesterol for stabilization, phospholipids to form a lipid bilayer structure, a lipid-linked polyethylene glycol that helps prolong the half-life of the composition and, most importantly, the ionizable cationic lipid, which improves the release of mRNA from the endosome to the cytoplasm [8, 37]. LNPs markedly prolong and improve protein expression *in vivo*, particularly after intradermal injection [40]. Application of exogenous RNA combined with a polymeric carrier was shown to activate the innate immune system by generating a local immunostimulatory environment [41], which consecutively also modulates adaptive immune responses. Last, the cationic peptide and immune activator protamine can be used as a carrier [42] as well as physical methods such as the gene gun, a microprojectile method [43], however the latter with only limited access in humans.

### Effects of exogenous mRNA on innate and adaptive immunity

mRNA is a transient copy of the coding genomic information and can be used for the expression of any therapeutic protein [44]. After the injection of an mRNA vaccine, the encoded protein will be translated and presented to the immune system. This process tightly resembles the natural course of a viral infection and its consecutive induction of a protective immune response. Once the exogeneous mRNA enters the cytoplasm, it will be processed similarly as endogenous mRNA. Hence, delivery of exogeneous mRNA to the cytoplasm is essential for antigen expression, but whether this is mediated through endosomal uptake and/or direct entry through the plasma membrane is not entirely clear [45].

For the efficient induction of adaptive immune responses, the translated candidate antigens need to be presented via MHC class I (MHC-I) and MHC class II (MHC-II) molecules on APCs, mainly DCs [46–48]. APCs own the unique ability to cross-present extracellular antigens (which are normally presented via MHC-II to CD4+ T cells) on MHC-I to CD8+ T cells [46, 48, 49]. The resultant cytotoxic T lymphocyte (CTL) induction is termed cross-priming [46]. Optimal CTL cross-priming further requires, as stated above, cross-talk to CD4+ T cells, which can recognize peptides presented via MHC-II [50, 51]. Of note, all nucleated cells can potentially process mRNAs and present derived peptides on MHC-I, but only APCs are able to present peptides on MHC-I and MHC-II, which is important for the induction of CD4+ T cell and B cell as well as antibody responses.

For efficient T cell activation several additional signals are essential. Next to antigen recognition, a costimulatory signal is required to induce the immune response [52] followed by consecutive cytokine production. Naturally, DCs express these costimulatory signals (such as B7 molecules) after sensing pathogen-associated molecular patterns (PAMPs), indicating microbial infection or danger [53]. Pharmacologically, this can be achieved by exploiting Toll-like receptor (TLR) ligands [54, 55]. TLRs belong to the group of pattern-PPRs, which are inherent to the innate immune system and whose function is the detection of PAMPs. TLRs are therefore located at different potential pathogen entry sites in the cell, e.g. the plasma membrane or endosome. Induction of IFN1 by viruses or other pathogens is indispensable for innate immune responses, confers anti-microbial activity [56] and is mediated via the activation of PAMPs [57].

**Fig. 1:**
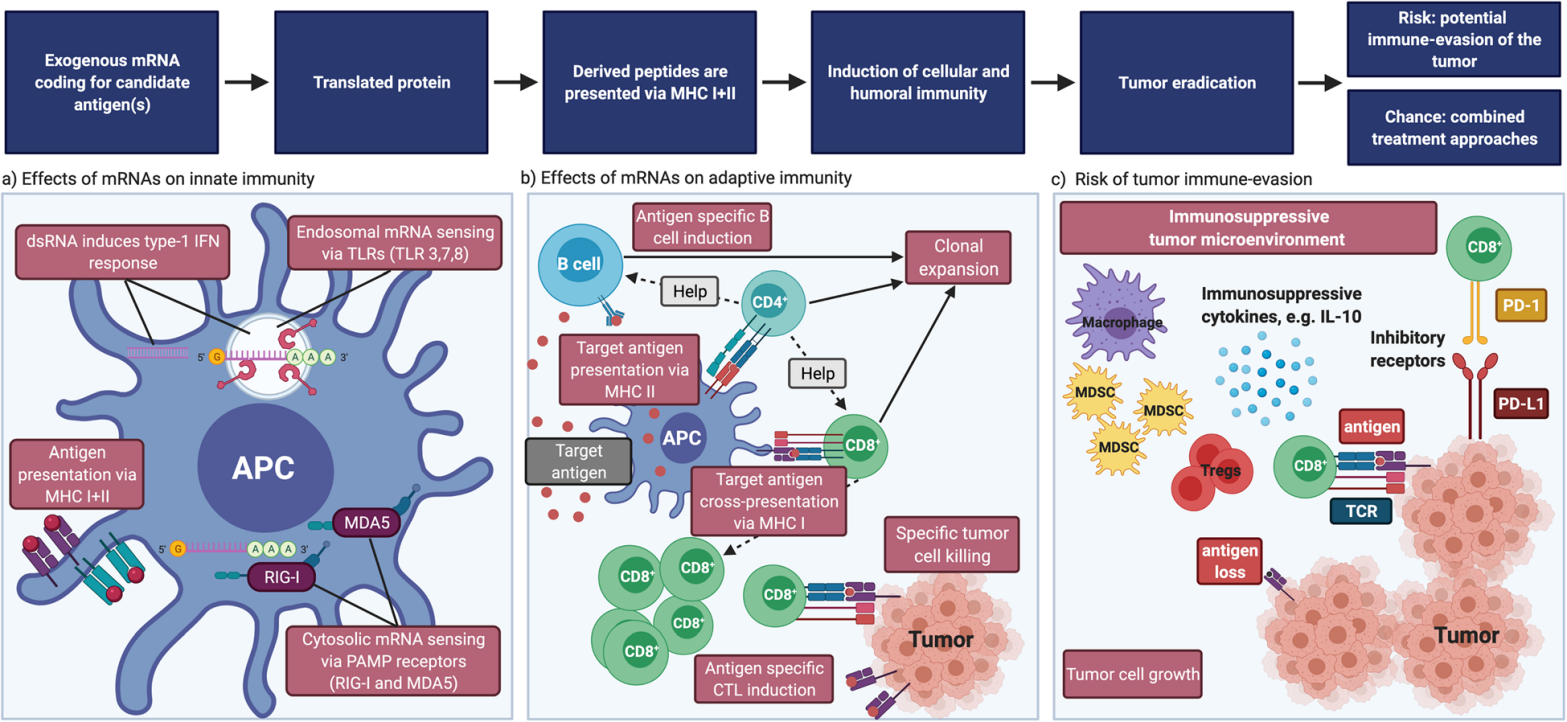
Effects of mRNA vaccines on immunity. **a** Effects of exogeneous mRNA on innate immunity. Exogeneous mRNA can be sensed by TLRs in the endosomes as well as receptors like RIG-I and MDA5 in the cytosol. dsRNA can induce a strong IFN1 response. Peptides derived from the translated protein will be processed in the proteasome and presented on MHC-I and MHC-II molecules. **b** Effects of exogeneous mRNA on innate immunity. APCs can present exogeneous antigens on MHC-II to CD4+ T cells and cross-present on MHC-I to CD8+ T cells. CD4+ T cells provide help to B cells and CD8+ T cells. Finally, clonal expansion of antigen-specific B and T cells results in target cell elimination. **c** Risk of tumor immune-evasion. Tumors are capable of creating an immunosuppressive micro-environment by recruiting myeloid-derived suppressor cells (MDSCs), regulatory T cells, M2 macrophages and the production of immunosuppressive cytokines. Upregulation of exhaustion markers on T cells, or antigen loss on tumor cells can further drive immune-evasion, exemplarily. CPI might help to regain immunosurveillance. BioRender was used to create the figure

It is important to highlight that exogenous mRNA is generally considered immunostimulatory, as it can activate innate immune cells via TLRs [8, 58], in particular TLR3, TLR7 and TLR8 [59, 60] (Fig. 1a). As soon as PRRs sense PAMPs an innate inflammatory response (including IFN1) is initiated with the consecutive activation of the adaptive immune response [61]. More precisely, TLR ligation leads to the production of proinflammatory cytokines, like TNF-α, IFN-α, IL-6, interferon-γ-induced protein 10 (IP-10) and the induction of costimulatory molecules, especially on APCs such as DCs [45, 62]. This finally results in the generation of adaptive B and T cell responses [61, 63]. TLR7 is expressed - amongst others - by B cells [64], macrophages and DCs [60, 65, 66] and can detect ssRNA [45]. In consequence, B cells are rapidly activated via the MYD88/TLR7-dependent signaling pathway and therefore provide stimuli for the regulation of adaptive immune responses induced by mRNA vaccines [45]. Moreover, TLR7 signaling augments production of proinflammatory cytokines, increases antigen presentation and improves memory B cell survival [45, 67].

In non-immune cells the cytoplasmic RIG-I-like receptors RIG-I and MDA5 sense exogenous RNA and mediate cytokine and chemokine production [24, 62, 68, 69], which in turn recruits innate immune cells such as DCs and macrophages to the site of mRNA injection [68] (Fig. 1a). Although this early induced robust cytokine production is warranted to enhance vaccine efficacy, different approaches in mRNA technology are pursued to minimize IFN1 induction, because of serious systemic side effects, such as autoimmunity. As an example, Miao and colleagues [70] have proposed that mRNA formulations exploiting an unsaturated lipid tail, a dihydroimidazole linker and cyclic amine head groups confer profound activation of APCs via the intracellular stimulator of interferon genes (STING) pathway, rather than through TLRs, thereby reducing systemic cytokine expression and increasing anti-tumor efficacy. Taken together, innate sensing of exogeneous RNA might confer stalled translation, degradation of RNA as well as consecutive minor antigen-specific immune responses [9], pointing at the close communication between innate and adaptive immunity after mRNA vaccination.

In order to induce adaptive immunity, specific antigens need to be presented to immune cells (Fig. 1b). mRNAs for cancer vaccines typically encode tumor-associated antigens (TAAs) that are preferentially expressed on cancer cells. To class these tumor-derived antigens, they can be subdivided into: i.) tissue differentiation antigens (e.g. CEA or MART-1, which can also be expressed on healthy tissues), ii.) tumor germline (cancer testis) antigens (e.g. NY-ESO-1 or MAGE-3), iii.) normal proteins overexpressed by tumor cells (e.g. EGFR, Muc-1, Her2/neu), iv.) viral proteins (e.g. EBV, HPV), and v.) tumor-specific mutated antigens (e.g. Mum-1, β-Catenin or CDK4) [6, 71, 72]. Genetic abnormalities are essential drivers for tumor development [73–75]. Somatic mutations may lead to the generation of neoepitopes, which are tumor-derived peptides [76] that bind to the MHC and thus can be recognized by autologous T cells. These neoepitopes are considered optimal cancer vaccine candidates [73]. In line, it has been demonstrated that neoepitope-specific T cells mediate clinical responses after being adoptively transferred or after immune checkpoint inhibition [77–80]. It is therefore not surprising that genetic cancer profiles containing a high burden of neoepitopes predict a better response to CPI, at least for melanoma, non–small cell lung cancer (NSCLC), and mismatch repair–deficient colorectal cancers [81].

While peptide-based vaccination approaches require to pick one single antigen restricted to a defined HLA molecule, mRNA vaccines allow the combination of mRNAs encoding different antigens. It was shown that mRNA-electroporated human DCs exhibited several MHC-I and II-restricted peptides and induced a polyclonal CD4+ and CD8+ T cell response [82]. Of note, CD4+ T cell help has been shown to be important for efficient induction of CTL and B cell responses [50, 51] and the presence of helper epitopes in the applied mRNA can further improve the immune response. Interestingly, the induction of CD4+ T cell responses after cytosolic delivery of mRNA into DCs was shown to be mediated by autophagy [83]. Furthermore, there is no clear size limitation for the encoded protein as mRNAs up to a length of 12 kilo base pairs have been used [37]. Last, mRNAs encoding for immunoregulatory proteins can be included in the vaccine composition, further improving its efficacy [84]. Taken together, vaccines including two or more mRNAs encoding for different proteins or long peptides can enable a broad and polyclonal immune response. Restrictions to certain HLA molecules and the risk of immune escape due to antigen loss are avoided. Upon application of mRNA coding for epitopes deduced from mutated proteins, a strong antigen-specific CD8+ T cell response is generated. Further, an efficient and durable CD4+ T cells-mediated tumor regression can be induced in vaccinated individuals. Kreiter and co-workers [85] demonstrated that the majority of tumor-specific mutations (the “mutanome”) is recognized by CD4+ T cells which confer strong antitumor activity. This CD4+ T cell response consists primarily of a robust T helper 1 (Th1)–biased immune response alongside with IFN-γ production by CD4+ and CD8+ T cells [85–87]. In regard to this strong Th1 response, different groups even tried to utilize mRNA vaccines as tools to modulate Th-polarization. An example for type 2 responses (which are characterized by secretion of the cytokines IL-4, IL-5, IL-13 and allergen-specific IgE) that are distinctive for allergies, mRNA vaccines were applied in order to reduce allergic T helper 2 (Th2) reactions [86].

Besides inducing sufficient T cell immunity, mRNA vaccines are further required to induce neutralizing antibodies, especially when targeting microbes. T follicular helper (Tfh) cells are not only crucial to generate germinal center (GC) responses, but also drive immunoglobulin class switch, affinity maturation and durable B cell memory responses. Although the exact mechanisms of Tfh cross-talk are not entirely clear, these cells should be activated by mRNA vaccines in order to generate sufficient amounts of efficaciously working and durable neutralizing antibodies [88]. Pardi and colleagues [40] applied a nucleoside-modified purified mRNA encapsulated in lipid nanoparticles (mRNA-LNPs) encoding various viral surface antigens (of ZIKA virus, HIV and influenza) intradermally and found that these vaccines induced antigen-specific CD4+ T cell, B cell and plasma cell responses as well as potent neutralizing antibodies in mice and non-human primates. They concluded, alongside with others, that nucleoside-modified mRNA-LNP vaccines are highly efficient in inducing sustainable neutralizing antibody production and that the introduction of noninflammatory modified nucleosides into the mRNA is the essential prerequisite [40]. Of note, the way of vaccine administration determines the duration of antigen expression (intramuscular and intradermal longer versus shorter after systemic application) and sustained antigen availability resulted in high antibody titers as well as GC-B cells and Tfh responses [89].

### The location of vaccine administration impacts the induced immune responses

**Fig. 2:**
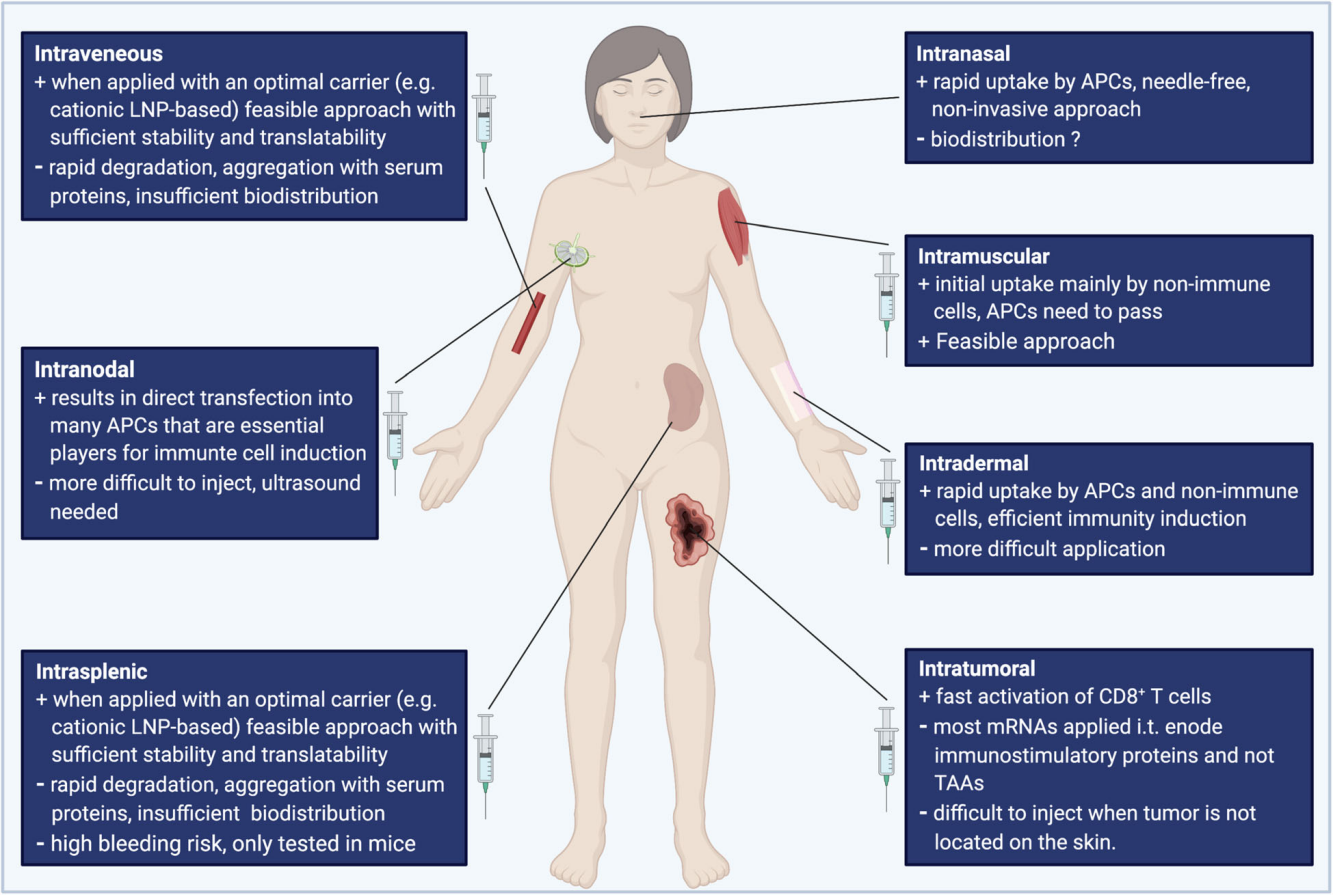
Different locations of mRNA injection can modulate the induced immune response. The advantages and disadvantages of different delivery ways are listed. BioRender was used to create the figure

In addition to the vaccine composition itself, also the way and location of delivery affects its efficacy [8, 37] (Fig. 2). Typically, vaccines are administered into the muscle or into the subcutaneous fat tissue. Besides this subcutaneous and intramuscular application, non-traditional routes such as intranodal, intrasplenic, intradermal, intranasal, intravenous and intratumoral mRNA applications exist and have been extensively analyzed for mRNA vaccination. Depending on its administration route, the vaccine will be taken up by very different cell types. The efficient delivery of the corresponding antigen to the draining lymph node is of high relevance. Secondary lymphoid organs are characterized by the tight presence of many immune cells, such as APCs and T cells, creating an ideal place for efficient induction of adaptive immune responses. DCs are a highly specialized, heterogeneous subset of APCs that link innate sensing of pathogens with the activation of adaptive immunity [90]. All DCs possess the unique ability to process and present antigens.

Roughly, DC can be devided into two major subsets in mice and men: i) plasmacytoid DCs (pDCs) and ii) myeloid/conventional DCs (mDCs) [91–95]. The latter can further be devided into myeloid/conventional DC1 (cDC1) and myeloid/conventional DC2 (cDC2).

While cDC1 are located in murine lymph nodes, in humans, XCR1 and C-type lectin domain 9 member A (CLEC9A) DCs are potentially essential for vaccine-induced immunity [50, 96]. Accordingly, if injected directly into a lymph node, due to the high amount of locally available immune cells, exogenous mRNAs will primarily be taken up via micropinocytosis by APCs generating CD4+, CD8+ T and B cell responses. The same holds true for *ex vivo* mRNA transfection of DCs followed by later injection. In line, several preclinical and clinical studies have used intranodal injection and proofed that this is a very efficient way of RNA delivery [97–99], also most likely due to close proximity to immune cells. Similar results were obtained for an intrasplenic application in mice [100].

Interestingly, also intranasal administration works similarly, namely through rapid antigen-uptake by DCs. The upper respiratory tract, including the nose, contains mucosal tissue which functions as physical barrier and first line of defense against invading pathogens [101]. The second line of defense consists of immune cells, especially dendritic cells, which form extensive networks within the nasal epithelium [101]. Data from studies with patients suffering from allergic rhinitis have proven that DCs are capable of opening tight junctions which enables them to access antigens beyond the epithelium [101, 102]. Using intranasal application, an LNPs-complexed mRNA vaccine reduced the onset of tumor development and augmented overall survival in murine tumor models of the OVA-expressing E.G7-OVA T-lymphoblastic cell line [103]. This way of delivery could be promising due to the advantages of its noninvasive, easy-to handle nature [8, 103].

In the human skin, many APCs are present, in particular Langerhans cells in the epidermis as well as interstitial DCs in the dermis [104]. Hence, after intradermal application, mRNAs are taken up and expressed locally at the injection site, by the many APCs in the skin [105], but also, even predominantly, by non-immune cells [44]. Probst and colleagues reported that these were mainly muscle cells, fibroblasts, and keratinocytes [44]. They demonstrated that intradermal injection of a globin UTR-stabilized (RNActive, CureVac GmbH) luciferase-encoding mRNA into the ear pinna of mice showed a luminescence peak after approximately 17 hours and was undetectable after 3 days [44]. Injection of the exogenous mRNA into human skin led to the expression of the expected protein. This study amongst others [106–111] provided the proof-of-concept that direct injection of RNA accommodates have sufficient stability and that it represents a feasible and efficient vaccination approach [44].

Only few immune cells are present in skeletal muscles [112, 113]. Next to the tissue-resident immune cells, circulating immune cells will eventually pass by and process and present the antigen at the site of intramuscular administration [112]. Of note, this local innate immune response and the magnitude of local inflammation will impact the consecutive adaptive immunity [112]. For this reason, traditional vaccines contain adjuvants that promote inflammation at the delivery site facilitating immune cell recruitment and activation [112].

Hence, after intramuscular vaccination of mRNA vaccines, mRNA will primarily be processed by local myocytes, but APCs will eventually pass through and induce antigen-specific CD8+ T cells.

Intratumoral injections have been assessed in several studies. To this end, the immune cell composition within a tumor is of high importance. Some so-called “hot” tumors are characterized by a high infiltration of immune cells and are associated with higher treatment responses and improved survival [114]. The magnitude of infiltrating immune cells therefore increases or limits the efficacy of currently available immunotherapies. Many approaches have been tested to switch “cold” to “warm” tumors. Lately, Newman and colleagues have even demonstrated that application of the seasonal influenca vaccine into a tumor facilitates the shift towards a warm tumor [114]. In line with this, direct injection into the tumor may enable a fast activation and expansion of possibly pre-existing antigen-specific T cells. However, many of these approaches use mRNAs encoding immunostimulatory proteins, and not TAAs [8, 66, 115]. Supporting this, the intratumoral application of TriMix mRNA not coding for TAAs led to the activation of CD8a+ DC, T cells and reduced tumor growth in various murine tumor models (E.G7-OVA, P815, A20, and TC-1) [116, 117].

Last, mRNAs can be injected intravenously, however several risks and hurdles have to be overcome. The aggregation with serum proteins and immediate degradation [8] necessities the packaging into carrier molecules in order to improve mRNA uptake, translatability and stability [8, 39, 118–120]. Moreover, the biodistribution of mRNA vaccines after intravenous injection is critical [8]. Exemplarily one study reported that their cationic LNP-based complexing vaccine mainly was detected in the liver after systemic administration [121]. Under certain conditions it therefore appears attractive to transport the translated protein into specific subcellular compartments via addition of targeting sequences, or by addressing specific receptors on DCs. Using a DNA (and here not RNA) vaccine, this could be achieved by using a DEC-205 fusion protein [122, 123]. Another very appealing approach is to target DCs using intravenously administered RNA-lipoplexes based on lipid carriers by optimally adjusting net charge [124]. It was previously shown that negatively charged particles resulted in trafficking of DCs into secondary lymphoid tissues and bone marrow where they induced robust IFN1 and a strong tumor-specific immune response [124]. The authors were the first to develop this novel class of systemically administered nanoparticulate RNA vaccines, which enable an optimal biodistribution to APCs into secondary lymphoid organs [124]. In contrast to other models, no functionalization of nanoparticles with molecular ligands that target DCs are necessary [8]. Instead, precise DC targeting is achieved using well-known lipid carriers and solely by adjusting negative net charge of the nanoparticles [124].

After systemic vaccine administration cells that engulfed the exogenous mRNA will circulate and eventually arrive in lymph nodes, where the induction and activation of immune responses can take place. In draining lymph nodes, activated immune cells such as B cells accumulate [68], help promote DC maturation by glycosylation-dependent interaction and boost antigen presentation [68, 125]. The increased production of proinflammatory cytokines such as TNF-α as well as the augmented expression of matrix metalloproteinases on the membrane of migrating DCs facilitates the trafficking of DCs toward the draining lymph nodes [126]. Lastly, the upregulation of activation markers such as CD69 on immune effector cells within the lymph nodes combined with the present pro-inflammatory cytokines will support effective immune priming [127].

Overall, these observations accentuate that direct transfection of APCs with mRNA is not an indispensable prerequisite for the effective induction of immune responses [45], and that systemic injections are, in principle, feasible. However, direct mRNA injection into secondary lymphoid tissue facilitates a targeted antigen delivery to APCs without the necessity for DC migration [8]. Intramuscular and intradermal vaccine administration results in a more persistent protein expression. In one study the half-life of mRNA-encoded firefly Luciferase was three times longer after intradermal in contrast to intravenous injection [121].

### Modulation of mRNA immunogenicity

While improved immune activation can be of interest in vaccination strategies and might even replace adjuvant application, innate immune sensing can also create an unfavorable environment for the translation of mRNA vaccines and thus limit antigen expression [45, 128, 129]. Some naturally occurring modified nucleosides have been reported to diminish TLR activation when incorporated into the transcript [63]. One such example are pseudouridines, which aim at suppressing RNA-mediated immune activation while improving the translational capacity and stability of the RNA [128]. Although this process significantly improves expression of intra- and extracellular proteins and reduces the immune response, a residual induction of IFN1 and proinflammatory cytokines remains [128]. In order to achieve high translatability and reduced RNA sensor activation, contaminants from IVT mRNA preparations have to be removed. Phage polymerase, which is used to generate mRNA, also generates multiple contaminants such as short RNAs or dsRNA. While short RNA moieties can be removed using polyacrylamide gel electrophoresis [130], contaminants in longer mRNA preparations can only be removed by chromatography, such as high performance liquid chromatography (HPLC) [129]. Removal of the latter is essential because dsRNA represents a potent PAMP that is sensed by PRRs as described above [8] and induces IFN1 production. This can, in turn, activate protein kinase R and 2′–5′-oligoadenylate synthetase and result in translation inhibition and RNA degradation [112, 131]. Hence, mRNA purification with HPLC facilitates excellent translatability without mediating IFN1 and proinflammatory cytokine responses [129]. In summary, efficient protein expression in DCs without deleterious systemic inflammation can be achieved by introducing modified nucleosides, complexing the mRNA with carrier molecules [107, 132] and applying purification methods such as polyacrylamide gel electrophoresis and HPLC [129].

While a detrimental systemic immune activation needs to be avoided, mRNAs coding for T cell molecules that mediate T cell activation and Th-1 responses can be exploited as components of the vaccines. One example is TriMix, which combines mRNAs encoding for three different immune-stimulating proteins, namely CD40 ligand (CD40L), CD70 and constitutively active TLR4 [8, 117, 133]. TriMix mRNA combinations have made their way into several vaccination trials due to improved DC activation and enhanced induction of CD8+ T cell responses [133].

Furthermore, the CureVac company has developed RNActive® vaccines with so-called self-adjuvant activity. They contain only naturally occurring nucleotides and are complexed with protamine [134, 135]. This co-delivered RNA particularly boosts the B and T cell response, including T effector and memory responses, and the expansion of subpopulations such as Th1 and Th2 cells and GC B cells [134]. In preclinical models RNActive® vaccines efficiently protected against different influenza strains and have shown anti-tumor effects [68, 69, 134–137].

In addition, cationic lipids, which are considered to increase RNA uptake and facilitate endosomal escape [138], can increase the adjuvant activity of mRNAs [139]. In line, the combination of a synthetic mRNA sequence with a polymeric carrier augments the adjuvanticity of distinct subunit vaccines [41].

mRNA co-delivered with cationic lipids induces a IFN1-mediated innate immune and CD8+ T cell responses and promotes cytolytic effector functions in murine tumor models. The absence of IFN1 and experiments in IFNAR^−/−^ mice [62] showed impaired efficacy of the vaccine [124] stating once more that the role of IFN1 is still not entirely clear in the context of mRNA vaccination. IFN1 has been associated with reduced exogenous RNA replication and expression and the promotion of T cell exhaustion [8]. Further studies investigating the detailed mechanism of action of IFN1 on the induced immune response will need to clarify these open questions. Nevertheless, it must be pointed out that immunogenicity was less pronounced in humans than it was expected according to most previous animal experiments [140, 141].

### Preclinical advances and clinical results of mRNA vaccinations against cancer

In contrast to prophylactic vaccination approaches for infectious diseases, cancer vaccines are usually applied in a therapeutic setting. They predominantly aim at inducing an efficient CD8+ T cell response against tumor-derived antigens that will stop or at least reduce growth of established tumors [142], while humoral immunity is probably less important. mRNA vaccination was first described as potential anti-tumor treatment by Conry and colleagues in 1995 [143] who constructed mRNA transcripts encoding luciferase and human carcinoembryonic antigen (CEA). The authors postulated that their strategy might be advantageous to induce an immune response to a proto-oncogene or growth factor that is generally associated with malignant transformation [143]. Almost contemporaneously, it was demonstrated that DCs transfected with mRNA encoding TAAs or total mRNA that was subcutaneously administered into tumor-bearing mice induced T cell immunity conferring growth inhibition of established tumors [144]. Gilboa and his co-workers were the first to demonstrate that DCs can utilize mRNA encoding TAAs to induce a protective anti-tumor immunity [145, 146]. Since then, an armamentarium of preclinical and clinical studies has been initiated and provided proof-of-concept that mRNA vaccination can efficiently induce immune responses that eliminate cancer cells. Novel approaches of delivery and complexing of the vaccine as well as increasing knowledge of mechanisms involved in innate and adaptive immune sensing have paved the way to these successful clinical trials. Different methods of mRNA vaccination against cancer have been pursued: basically, mRNA vaccines against tumors are either generated i) using *ex vivo* loaded or electroporated DCs or ii) by direct injection of mRNA with or without a carrier. When DCs are used, these first have to be isolated or *in vitro* generated and then transfected with the mRNA encoding the TAA(s) followed by re-transfusion into the patient. Transfection is usually performed using electroporation [147] and immunotherapy with mRNA-electroporated DCs was demonstrated to be safe in cancer patients [148, 149]. DCs electroporated with mRNA encoding ovalbumin or tumor-derived mRNAs generated strong tumor-specific immune responses [144] in different murine melanoma models and vaccination trials. The efficacy of these vaccines could be further augmented by the addition of mRNAs encoding immune regulatory proteins such as CD83 [150], 4-1BB ligand, cytokines or IL-12 [7, 84, 151], exemplarily. In line with this, electroporation of DCs with mRNAs together with the aforementioned immunostimulatory TriMix increased the efficacy of the vaccine and improved the induction of anti-tumor mediated immune responses in several pre-clinical studies due to upregulation of costimulatory molecules on DCs and an enhanced stimulatory capacity [117, 135, 152]. In addition, the generated CD4^+^ T cells showed a higher activation status and demonstrated a pronounced shift from Tregs to Th1-like phenotype [116, 117, 153]. In humans, vaccination with mRNA coding for melanoma-associated antigens combined with TriMix led to impressive tumor regression of advanced-stage melanomas [154]. These first reports were followed by several clinical trials applying DC-based mRNA vaccines in cancer patients (reviewed in [133, 155]) and are currently advancing to combined treatment approaches using vaccination in addition to chemotherapy, radiation or checkpoint blockade (Tables 1 and 2). Some of these studies have yielded durable tumor growth inhibition [154]. Although these DC-based approaches are efficacious due to the direct targeting of DCs as most powerful APCs, they are also complex and cost-intensive.

**Table 1:** Clinical results from studies using mRNA vaccines in tumor therapy (extract)

Mrna encoding for	Vehicle	Entity	Outcome	Reference
WT1	DC	AML	Induction/maintenance of CR. Clinical responses correlated with vaccine-associated increases in WT1-specific CD8^+^ T cells	NCT00834002 [156]
WT1	DC	Solid tumors	Unknown; in malignant pleural mesothelioma undefined clinical benefit, vaccine-elicited immunity was obtained in 9/10 pat.	NCT01291420 [157]
WT1	DC	AML	Prevent or delay relapse in 43% of patients with AML in remission after chemotherapy	NCT00965224 [158]
WT1/PRAME	DC	AML	Specific T cell responses in 4/5 patients; CR after 21,25,33 months in 3 pat.	[159]
WT1/PRAME/cmvpp65	DC	AML	2/7 pat. Exhibited responses to PRAME and WT each. 7/10 vaccinated pat. are still alive, and 5/10 are in CR, with an observation period of up to 840 d	*NCT01734304* [160]
hTERT	DC	AML	Maintenance of CR: 11 pat. (58%) developed hTERT-specific T-cell responses; median follow-up of 52 months, 58% of pat. In CR (11 of 19 patients) were free of disease recurrence	NCT00510133 [161]
Whole tumor RNA	DC	Pediatric brain cancer	2 of 7 pat. SD clinical and 1 of 7 showed a PR	[15]
Tumor RNA plus synthetic CD40L RNA	DC	Metastatic RCC	In combination with sunitinib: 13 pat. (62%) experienced clinical benefit (9 PR, 4 SD);	NCT00678119 [162]
NY-ESO-1, MAGE-A3, tyrosinase and TPTE	RNA-lipoplexes (iv)	Melanoma	Ifnα and strong antigen-specific T-cell responses were induced, SD/PR	[124]
MAGE-C1, MAGE-C2, NY-ESO-1, BIRC5, 5T4	Vaccine containing self-adjuvanted mRNA	NSCLC	Antigen-specific immune responses against ≥1 antigen were induced in 65% of pat. ≥ 2 fold increase of pre germinal center B cells; no objective clinical responses follow up trial terminated due to low recruitment	NCT01915524 [163]
PSA, PSCA, PSMA, and STEAP1	Vaccine containing self-adjuvanted mRNA	PCA	26/33 evaluable pat. Treated developed an immune response, directed against multiple antigens in 15 out of 33 pat. One pat. Showed a confirmed PSA response. In the subgroup of 36 metastatic patients, the Kaplan-Meier estimate of median overall survival was 31.4 months	EudraCT number 2008-003967-3 7[164]
Multiple TAAs	DC	PCA	The addition to of docetaxel to dcvac was safe. Immune responses were detected in approx. Half of the pat. No effect on PFS and DSS	[165]
Autologous tumor-mRNA	DC	Melanoma	A tumor-specific immune response was demonstrated in 16/31 pat. The response rate was higher after intradermal than intranodal vaccination (80% vs. 38%). Immune responders had improved survival compared to non-responders (median 14 mo vs. 6 mo; *p* = 0.030), and all 8 pat. Surviving >20 mo were immune responders.	NCT01278940 [166]
Cancer stem cell mRNA	DC	Glioblastoma	An immune response induced by vaccination was identified in all 7 pat. Compared to matched controls, progression-free survival was 2.9 times longer in vaccinated patients (median 694 vs. 236 days, *p* = 0.0018, log-rank test).	[167]
CEA	DC	CRC	All patients showed T-cell responses against the control protein (keyhole limpet hemocyanin) upon vaccination. CEA peptide-specific T-cells were detected in 8 /11 pat. In the peptide group, but in 0/5 patients in the RNA group.	NCT00228189 [168]
Tyrosinase and gp100	DC	Melanoma	One mixed tumor response and two durable tumor stabilizations were observed among 8 pat. With evaluable disease at baseline	NCT01530698 [169]
Total tumor RNA	Naked	Melanoma	Increase in antitumor humoral immune response was seen in some patients after i.d. Injection of naked mRNA. However, a demonstration of clinical effectiveness of direct injection of mRNA for antitumor immunotherapy was not shown in this study and must be evaluated in subsequent trials.	[106]
Melan-A, gp100, Tyrosinase, Mage-A1, Mage-A3, and survivin in 21	Protamine-stabil. mRNAs	Melanoma	A reproducible increase of vaccine-directed T cells was observed in 2/4 immunologically evaluable patients. 1/7 pat. With measurable disease showed a complete response. In conclusion, we show here that direct injection of protamine-protected mRNA is feasible and safe	NCT00204607 [170]
MUC1, CEA, Her2/neu, telomerase, survivin, MAGE-A1	I.d. mRNAs	Metastatic RCC	Median survival of 24.5 mo (all patients) and 89 mo (favorable risk patients). Long-term survivors displayed immunological responses to the applied antigens while no patient without detectable immune response had survived more than 33 mo.	[110, 111]
4 Melanoma associated non-Mutated antigens	I.v. mRNAs	Melanoma after CPI	Lipo-MERIT trial: melanoma fixvac, alone or in combination with CPI, mediated durable objective responses in melanoma pat. After CPI treatment. Responses are closely associated with strong CD4^+^ and CD8^+^ T cell responses against the vaccine antigens.	NCT02410733 [171]

Abbreviation: *RCC* renal cell cancer, *CRC* colorectal cancer, *PCA* prostata cancer

**Table 2:** Currently recruiting studies using mRNA vaccines in tumor therapy

mRNA encoding for	Vehicle	Entity	Concept	Reference
OX40L, IL-23, and IL-36γ	LNP	Dose escalation: r/r solid tumor or lymphoma Dose expansion: triple negative BC, HNSC, NHL and urothelial cancer	mRNA-2752 +/- durvalumab (phase 1)	NCT03739931
individually designed mRNA coding for tumor neoantigens	LNP	melanoma	mRNA4157 +/- pembrolizumab (phase 2)	NCT03897881
individually designed mRNA coding for tumor neoantigens	LNP	solid tumors: pat. with resected solid tumors and in combination with pembrolizumab in pat. with unresectable solid tumors	mRNA4157 +/-pembrolizumab (phase 1)	NCT03313778
KRAS G12D, G12V, G13D, and G12C	LNP	KRASmut NSCLC, CRC or PancCA	mRNA-5671/V941+/- Pembrolizumab (phase 1)	NCT03948763
NY-ESO-1, MAGE-C1, MAGE-C2, survivin, 5T4, and MUC-1	Protamine	NSCLC	mRNA vaccine BI 1361849 + durvalumab +/- tremelimumab (phase 1/2)	NCT03164772
CD70, CD40L, and constitutively active TLR4	Naked mRNA encoding DC activating proteins	Early breast cancer and accessible tumor lesions	TriMix vs placebo (phase 1)	NCT03788083
W_ova1 Vaccine (includes 3 OC TAA RNAs)	Liposome	Ovarian cancer	Only treatment arm mRNA + chemotherapy (phase 1)	NCT04163094
tyrosinase, gp100, MAGE-A3, MAGE-C2, and PRAME + CD70, CD40L, and constitutively active TLR4	LNP	Melanoma	ECI-006 intranodal injection with different doses and frequencies following surgical resection, and in patients with stable disease after standard of care immunotherapy treatment + anti-PD1 (cohort 2)	NCT03394937
Wt1	DC	AML	Autologous WT1 mRNA electroporated DCs during 2 years with repeated injection vs no intervention (phase 2)	NCT01686334
5 antigens expressed in de novo and metastatic prostate cancer	liposomes	PCA	W_pro1 in patients with metastatic castration resistant prostate cancer +/- Cemiplimab and/or goserelin acetate in patients with high-risk, localized prostate cancer	NCT04382898
survivin, hTERT and autologous tumor stem cells derived from tumorspheres	DC	Glioblastoma	Trivalent (temodal/irradiation +-vaccination) in primary treated patients with IDH wild-type, MGMT-promotor methylated glioblastoma (phase 1/2)	NCT03548571
PD-L1/L2-silenced, MiHA mRNA	DC	Hematological malignancies after allo HSCT	Vaccination with PD-L1/L2-silenced minor histocompatibility antigen-loaded donor DC vaccines to boost graft-versus-tumor immunity after allogeneic stem cell transplantation (a Phase I/II Study)	NCT02528682
WT1	DC	Malignant pleural mesothelioma	DC vaccination + chemotherapy	NCT02649829
WT1	DC	Glioblastoma	Newly diagnosed glioblastoma when autologous WT1 mRNA-loaded DC vaccination is added to adjuvant temozolomide maintenance treatment following (sub)total resection and temozolomide-based chemoradiation	NCT02649582
pp65-lysosomal-associated membrane protein	DC	Glioblastoma	pp65-shLAMP mRNA DCs with GM-CSF + Td or pp65-flLAMP mRNA DC with GM-CSF + Td or unpulsed PBMC and saline + temodal (phase 2)	NCT02465268
pp65-lysosomal-associated membrane protein	DC	Glioblastoma	Human CMV pp65-LAMP mRNA pulsed autologous DCs Preconditioning with unpulsed DC vs. Td toxoid vs. varlilumab	NCT03688178
Total tumor mRNA	DC	Glioblastoma	TMZ during RT and TTRNA pulsed DC´s +Td+GMCSF during and after maintenance cycles of dose-intensive TMZ vs. focal radiotherapy alone and TTRNA pulsed DC´s +Td+GMCSF without maintenance DI TMZ	NCT03396575
Up to 20 neoantigens (individualized)	LNP	Solid tumors	RO7198457 +- Atezolizumab (phase 1)	NCT03289962
Up to 20 neoantigens (individualized)	LNP	Melanoma	RO7198457 +- Pembrolizumab (phase 2)	NCT03815058

In contrast, direct injection of naked or complexed mRNA is considered to be a fast and feasible approach. A variety of studies in different tumor entities has proven that direct RNA injection is efficacious and that the RNA is not degraded by RNAses before an efficient translation into a protein can take place (Tables 1 and 2). Exemplarily, a phase I/II trial tested the repeated intradermal application of an mRNA vaccine encoding six different TAAs (MUC1, CEA, Her2/neu, telomerase, survivin, MAGE-A1) in 30 metastatic RCC patients [111]. Long-term results after 10 years stated that mRNA vaccination is safe and efficacious [110]. It delayed tumor growth and increases survival, which was tightly associated with the detected immune responses against the TAAs [110]. Another study investigated an intravenously administered liposomal-complexed RNA (RNA-LPX) vaccine (melanoma FixVac BNT111) encoding four non-mutated, melanoma-associated antigens in patients with advanced, unresectable melanoma after CPI therapy [171]. The authors clearly demonstrated that the vaccine alone or in combination with CPI mediated durable objective responses in the included patients and that the responses were closely associated with strong CD4^+^ and CD8^+^ T cell responses against the vaccine antigens [171].

To name two last examples, seven patients with locally advanced and 39 patients with metastatic NSCLC received five intradermal applications of CV9201, an RNActive-based vaccine encoding five NSCLC antigens (NY-ESO-1, MAGE-C1/2, survivin, trophoblast glycoprotein (5T4)) [172]. 63% of these patients developed antigen-specific immune responses against at least one antigen and 60% showed an increase of activated IgD+ CD38^high^ B cells. 31% of these patients had stable disease, while the other two third progressed [172]. A similar study aiming to improve anti-tumor immunity examined vaccination with CV9202 combined with local radiation in patients with advanced NSCLC [173]. CV9202 is an RNActive-based vaccine that encodes six NSCLC TAAs (NY-ESO-1, MAGE-C1, MAGE-C2, 5T4, survivin, and MUC-1). Antigen-specific cellular and humoral immunity was increased in contrast to baseline in the majority of patients. One patient treated with the vaccine, radiation and chemotherapy experienced a partial response (PR), 46.2% showed a stable disease (SD) [173].

### Strategies to improve vaccine-induced anti-cancer immune responses

Most cancer vaccine trials have limited success in patients with advanced disease or therapy-refractory tumors [174, 175]. Most commonly, efficient T cell induction is the major hurdle. TAAs are non-mutated self-antigens which commonly induce central T cell tolerance [176].

A variety of techniques exist that ameliorate mRNA delivery, composition, immunogenicity or translatability in general. However, several specific considerations for immune responses against tumors need to be considered. These include the optimal timing of vaccine application (adjuvant vs. advanced stage disease), the choice of the target antigen, the manipulation of the tumor micro-environment and the potential combination with other cancer treatments that might cause additive or even synergistic anti-tumor effects.

Anti-cancer vaccines are usually applied when the tumor has reached a reasonable size or has already metastasized. These cancer cells already interacted for a longer time with the immune system and may have developed cell-intrinsic or extrinsic mechanisms that help them to escape from the immune system and diminish the therapeutic effect of the vaccine-induced anti-cancer immunity [177]. mRNA application in earlier stages of disease, such as during adjuvant therapy could therefore improve the efficacy of vaccine treatments. One recent study analyzed in a randomized phase II clinical trial the effect of a treatment with autologous DCs co-electroporated with mRNA coding for TriMix and mRNA encoding one of four TAAs (MAGE-A3, MAGE-C2, tyrosinase, or gp100) linked to an HLA class II targeting signal [152, 178]. These melanoma patients were not allowed to have any evidence of disease following the resection of macro-metastases. This adjuvant intradermal/intravenous treatment was well-tolerated and improved the 1-year disease-free survival rate [152]. The authors pointed out that a cotreatment with CPI or a targeted therapy might further improve survival of patients with high risk of recurrence [152].

The interindividual heterogeneity between cancer patients can hinder efficient immunity induction and significantly differentiates the immune response against a microbe or against a tumor. Tumors accumulate up to thousands of genomic mutations of which only a fraction results in the generation of novel epitopes that can be recognized by the immune system and confer inhibition of tumor growth [179]. Advances in next generation sequencing (NGS) technology allows to decipher the genome, exome and transcriptome of an individual cancer patient [12, 180]. This new-gained knowledge about the heterogeneity of different tumors and neoepitopes of specific T cells has greatly advanced the progress of personalized cancer therapy. In 2012, the Sahin group proposed that the “mutanome” could be exploited for tumor vaccination [180, 181]. For a first preclinical trial of “mutanome engineered RNA immunotherapy” (MERIT) the C57BL/6-derived B16F10 melanoma cell line was used, which has in contrast to healthy cells several hundreds of targetable mutations. Amongst these, 16 of 50 were recognized by T cells and only a fraction of these were associated with cancer growth in vaccinated mice [181]. A first-in-human clinical study followed on the basis of these promising results and aimed to target multiple immunogenic tumor mutations of each individual patient. The group applied NGS-based mutation identification coupled with bioinformatic target selection for prediction of an effective therapeutic vaccine [10]. The advantage of this or similar approaches is the personalized selection of individual immunogenic mutations of a patient´s tumor for the construction of a personalized, unique vaccine containing RNAs that encode mutation-coding sequences [10, 182–184].

The cross-talk of the tumor microenvironment with immune and non-immune cells can significantly impact tumor cell growth. Most tumors strive to evade the immune system by expressing immunosuppressive proteins on the surface (e.g. programmed death ligand 1 (PD-L1), osteoactivin), by producing immunosuppressive cytokines (e.g. IL-10) and chemokines and by recruiting immunosuppressive cells such as regulatory T cells, M2 macrophages and myeloid-derived suppressor cells (Fig. 1c). This hostile tumor microenvironment results in anergy and exhaustion of T cells promoting prolonged tumor cell survival [177, 185, 186]. Thus, an ideal tumor vaccine would improve the local immune cell composition and restore tumor immunosurveillance [110, 177]. CPI have been shown to impact on the architecture of the tumor microenvironment and exhibit the potential to reinvigorate and expand pre-existing T cells or induce “new” anticancer immune responses. Of note, a high infiltration with (pre-existing) CD8+ T cells is associated with an improved outcome and a higher chance to respond to CPI [177, 187, 188]. It has further been shown for mRNA vaccination approaches that the survival across different cancer types correlates with the induction of T cells against predicted neoepitopes [189]. These neoepitope-specific T cells are tightly associated with the response to CPI treatment [190, 191]. CPI such as monoclonal antibodies against cytotoxic T lymphocyte antigen 4 (CTLA-4), programmed death 1 (PD-1) and PD-L1 are able to at least partly remove immunosuppression [177, 188, 192]. Generally, CPIs are capable of maintaining a once induced immune response and prevent up-regulation of T cell exhaustion markers making them a promising combination partner for mRNA vaccines [193]. Exemplarily, a combination of CPI with an mRNA vaccine encoding TRP2 elicited a robust TAA-specific immune response in a C57BL/6 mouse model of B16F10 melanoma leading to inhibition of tumor growth [193]. The co-delivery of PD-L1 siRNA and mRNA vaccine in this model downregulated PD-L1 in antigen-presenting DCs resulting in increased T cell activation and proliferation [193]. In line with this work, many of the currently recruiting mRNA trials against cancer use protocols that combine mRNAs together with CPI (shown in Table 2 (grey shaded)). Hence, neoantigen-targeting immunotherapies, preferentially in combination with checkpoint blockade, but also radiation or chemotherapy might change the landscape of anti-cancer treatments. Finally, additional options for improving patient´s survival could be achieved by utilizing combinatorial approaches that include specific adjuvants or activating monoclonal antibodies (4-1BB, ICOS, CD40) in the therapeutic regime [177, 194] .

### mRNA vaccine development against microbes in the context of the ongoing SARS-CoV2 pandemic

Vaccine development has been in the spotlight during the ongoing SARS-CoV2 pandemic. Never before has such a tremendous audience participated in every single step of vaccine design. Although mRNA was historically considered instable and immunogenic, mRNA vaccines have become the favored treatment option for infectious diseases. This is mainly due to their versatile nature, their safety as well as relatively simple and fast manufacturing process. The latter, in particular, becomes important in a worldwide pandemic, where short development and design times and a rapid production of millions of vaccines under good manufacturing practice conditions are indispensable. mRNA vaccines against infectious diseases have been shown to induce strong CD4+ and CD8^+^ T cell responses [8, 134, 195, 196] as well as neutralizing antibodies after only one or two low-dose immunizations in animals [134, 197–202].

Concerning the way of delivery, mRNA-based DC vaccines can be generated through *ex vivo* loading of DCs and have demonstrated efficient T and B cell induction in HIV and CMV trials [37, 203–209]. However, *ex vivo* loading of DCs is time-consuming and expensive and therefore not suitable as rapid “off the shelf” vaccination during a pandemic. In another approach, mRNAs are designed to amplify themselves. Generally, these self-amplifying mRNA vaccines are based on a virus, such as the alphavirus [210]. Alphaviruses can, like many other viruses, mediate functions that antagonize the IFN response. This viral approach ensures that all the genes encoding the replication machinery of the RNA remain untouched, while the genes encoding the structural proteins of the virus are replaced by the target antigen [8]. The efficacy of these vaccines is further improved by RNA-complexing agents and the fact that many self-amplifying mRNAs display adjuvant activity themselves [8]. Although this adjuvant activity is rather appreciated in terms of improved immune responses, it is also considered critical due to potential uncontrollable immune reactions, which may limit its repeated use.

Direct injection of non-replicating mRNA is an attractive vaccination approach also against infectious diseases. This method is as aforementioned cheaper and easy to perform especially in settings with limited resources [8]. To name only a few examples of mRNA studies exploiting direct injection, the intradermal application of uncomplexed mRNA encoding several influenza virus antigens together with a protamine-complexed RNA adjuvant was shown to be efficient and protected mice from lethal virus challenge [137]. Intravenous vaccination of mice with lipid-complexed mRNA encoding influenza virus HA activated T cells after one single injection [124]. In line, a LNP-complexed nucleoside-modified non-FPLC-purified mRNA vaccine against influenza HA 10 neuraminidase 8 (H10N8) and H7N9 influenza virus strains proved to generate protective immune responses in several mammals and humans [37, 140, 141]. Efficient inhibition of haemagglutination and production of neutralizing antibodies was observed here. General side effects included local reactions at the injection site such as redness, pain and swelling. Systemic symptoms covered headaches, fatigue, chills and cold-like symptoms [140]. Alberer et al. recently demonstrated a first-in-human proof-of-concept clinical trial in healthy adults using a prophylactic mRNA-based vaccine encoding rabies virus glycoprotein, which induced humoral immunity when administered with a needle-free device [141]. Taken together, naked mRNA injection seems to be a feasible and broadly applicable approach for the protection against infectious diseases. As mentioned above, vaccine quality was improved by nucleoside modification or complexed mRNAs, and further shaped/influenced by the choice of delivery route and format, such as LNP vaccines, specific formulation components and the sequence selection.

Although no mRNA vaccine had been approved until end of 2020, the above listed developments paved the way for the rapid design of the recently introduced COVID19 vaccines. They were developed by several biotech companies and most of them use mRNA compositions. As of December 2020, a total of two mRNAs vaccines from BioNTech/Pfizer and Moderna are approved. BioNTech/Pfizer compared in their initial trials several RNA-based COVID19 pandemic vaccine candidates in clinical studies in Germany and the US [211–213]. Not all technical details of the vaccine compositions have been published yet, but it is known that they use LNP–formulated nucleoside-modified RNA [213]. The most promising vaccine candidates were BNT162b1 which encodes the SARS-CoV-2 receptor–binding domain [213] and BNT162b2 which encodes a modified version of the SARS-CoV-2 full-length spike protein [212, 213]. This modification helps to mimic the intact virus and aims at improving virus-neutralizing antibody responses [212]. BNT162b1 induced strong CD4+ T helper (Th1) cell responses and CD8+ T cells that efficiently produced IFNγ and IL-2 [11], thus providing both humoral and cell-mediated antiviral immunity. BNT162b2 was reported to show a good balance of efficacy and safety at the relatively low dose of 30 μg [211, 213] and therefore advanced to the international phase 2–3 clinical trials [212, 213]. About 44,000 adults were subjected to two intramuscular injections of 30 μg of BNT162b2 21 days apart (NCT04368728) [212] This regimen conferred 95% protection against Covid-19 [212]. The titers of SARS-CoV-2–neutralizing antibodies resembled or exceeded those found in patients that had recovered from a SARS-CoV-2 infection [11, 211–213]. Of note, the immunogenicity of the vaccine decreased with age, which is not surprising and most likely associated with immunosenescence [213, 214]^.^

In another, very recent study, BNT162b2 protected macaques from SARS-CoV-2 challenge, indicating that the vaccine not only induces potent cellular and humoral immune responses, but also avoids development of a severe course of disease [215].

Although the efficient induction of humoral and cell-dependent immunity by these vaccines is encouraging, many open questions remain. We are currently just beginning to understand the role of the immune system in COVID19, especially during a severe course of disease. We do not know, yet, how long a vaccine-induced immune response will persist and how efficient it will protect against COVID19 in general and against potentially arising mutations. Future trials will have to address these important questions. The clinical and immunological results from these trials will also have a profound impact on the improvement of vaccines designed against malignant cells.

### Safety aspects

Until 2020, no single mRNA vaccination had been approved in the world. Since the approval of different mRNA vaccines against SARS-CoV2 in 2020 amidst the ongoing pandemic, safety aspects become increasingly present. mRNA vaccines are free of cellular or animal components, have generally been shown to be safe and well-tolerated and display only few serious issues of concern. However, clinical experience on acute and long-term side effects are limited. In some cases, local reactions such as pain and redness at the injection site or systemic allergic reactions might occur. An integration into the patient´s genome is not possible and microbial contaminations are due to the short production process and storage at low temperatures extremely unlikely. Beside these local and systemic inflammatory reactions, the theoretical risk of uncontrollable inflammation and autoimmunity exists. This would mainly be mediated by the induced IFN1 response as described above [37, 62, 69, 199, 216]. Currently there is no clear evidence that induction of immunity against mRNA itself takes place. However, in patients with systemic lupus erythematosus and other autoimmune diseases it was proposed that development of anti-self RNA antibodies might trigger and progress autoimmunity [9, 217]. Besides that, a residual risk of toxic side effects associated with the delivery compounds, complexing agents as well as potentially inserted nucleotides remains. For the latter, it has to be considered that these side effects might only occur after a prolonged time after treatment [9].

### Future directions

The ongoing SARS-CoV2 pandemic has highlighted how fast and efficaciously mRNA vaccines can be produced against a newly arising threat. However, while vaccines against infectious diseases are usually applied in a prophylactic setting against well-defined antigens, most anti-tumor vaccines are administered when the tumor has progressed. Moreover, cancer target antigens display a high interindividual heterogeneity, contain a limited number of cancer-specific cell surface antigens and are less well characterized. Strategies to overcome immune escape mechanisms for improving vaccine efficacy should include combinatorial approaches with specific adjuvants, CPIs, T cell activating monoclonal antibodies or the modulation of the tumor microenvironment including cytokines, radiation or chemotherapies. mRNA vaccines could be applied together with T cell therapies such autologous TCR transgenic T cells or CAR-T cells and with bispecific antibodies (BITEs). As reported previously, the expensive manufacturing process of BITE production can be circumvented using a pharmacologically optimized, nucleoside-modified mRNA encoding the antibody [218]. This mRNA vaccination helped to eradicate large tumors in mice [218]. In addition, mRNA vaccines are considered to serve as tools for the transient modulation of immune cells [9, 171]. mRNA vaccines can not only encode specific antigens, but can also be used for the transient expression of antigen-specific TCR or CARs. If these mRNAs are transfected into immune cells such as T or natural killer cells, transfected cells can recognize and eliminate tumor cells expressing the targeted antigen. Exemplarily, mRNA was applied in one study to transiently force T cells to express an epidermal growth factor receptor (EGFR)-specific CAR to eradicate EGFR-expressing tumor cells [15].

While CAR T cells haven proven efficacious in B-cell malignancies, only very limited success has been observed in solid cancers, most likely due to the non-persistence of adoptively transferred CAR T cells and the limited disposability of tumor-derived antigens. Reinhard et al. exploited tight junction protein claudin 6 (CLDN6) as a CAR target in solid tumors [16]. The group found that CLDN6 as a transmembrane protein involved in tight junction formation is broadly expressed in fetal organs and a variety of solid cancers, but not in healthy tissues [16, 219]. They designed a second generation CLDN6-CAR equipped with a 4-1BB costimulatory domain. This CAR combined with intravenously applied liposomal antigen-encoding RNA (RNA-LPX) [124] for T cell stimulation resulted in delivery to all lymphoid organs [16]. This CAR-T cell Amplifying RNA Vaccine (CARVac) elicited an IFN1-driven immune response resulting in the induction and clonal proliferation of antigen-specific T cells, hence providing evidence for a possible administration of CARVac to tune the expansion of engineered T cells [16]. Most impressively, these T cells showed improved engraftment and reduced the growth of tumors in several murine tumor models [16].

In summary, the here described developments have paved the way for a promising future for mRNA vaccines and might contour the landscape of future anti-cancer treatments. Especially the importance of combinatorial concepts will advance future anti-cancer therapy approaches.

## Data Availability

Not applicable.

## Abbreviations

APC: Antigen presenting cells
ARCA: Anti-reverse cap analogs
BITEs: Bispecific antibodies
CAR: Chimeric antigen receptors
CarVac: CAR-T cell Amplifying RNA Vaccine
CPI: Checkpoint inhibitor
CEA: Carcinoembryonic antigen
CLDN6: Tight junction protein claudin 6
CTL: Cytotoxic T lymphocyte
CTLA-4: Cytotoxic T lymphocyte antigen 4
DC: Dendritic cell
ds: Double-stranded
GC: Germinal center
H10N8): Influenza HA 10 neuraminidase 8
IFN1: Type-1 interferon
IP-10: Interferon-γ-induced protein 10
IRES: Internal ribosome entry sites
IVT: *In vitro* transcribed
LNPs: Lipid nanoparticles
m5C: 5-methylcytidine
m5U: 5-methyluridine
m6A: N6-methyladenosine
m7G: 7-methylguanosine
MDSCs: Myeloid-derived suppressor cells
MHC-I: MHC class I molecule
MHC-II: MHC class II molecule
mRNA: Messenger RNA
NSCLC: Non–small cell lung cancer
ORF: Open reading frame
PAMPS: Pathogen-associated molecular patterns
PAP: Poly(A) polymerase
PD-1: Programmed death 1
PD-L1: Programmed death ligand 1
PR: Partial response
PRR: Pattern recognition receptors
RNA-LPX: Liposomal-complexed RNA
s2U: 2-thiouridine
SD: Stable disease
STING: Stimulator of interferon genes
TAAs: Tumor-associated antigens
TCR: T cell receptors
Tfh: T follicular helper cell
Th1: T helper 1
Th2: T helper 2
TLR: Toll-like receptor
Tregs: Regulatory T cells

## References

[1] Hannah Ritchie and Max Roser. Causes of Death - Our World in Data. . Available from: https://ourworldindata.org/causes-of-death. [cited 2020 Dec 1].

[2] Couzin-Frankel J (2013). Breakthrough of the year 2013. Cancer immunotherapy. Science.

[3] Hochhaus A, Larson RA, Guilhot F, Radich JP, Branford S, Hughes TP (2017). Long-term outcomes of imatinib treatment for chronic myeloid leukemia. N Engl J Med..

[4] Bringmann A, Held SAE, Heine A, Brossart P (2010). RNA vaccines in cancer treatment. J Biomed Biotechnol..

[5] Farhood B, Najafi M, Mortezaee K (2019). CD8+ cytotoxic T lymphocytes in cancer immunotherapy: A review. J Cell Physiol..

[6] Haen SP, Löffler MW, Rammensee H-G, Brossart P (2020). Towards new horizons: characterization, classification and implications of the tumour antigenic repertoire. Nat Rev Clin Oncol..

[7] Bontkes HJ, Kramer D, Ruizendaal JJ, Kueter EWM, van Tendeloo VFI, Meijer CJLM (2007). Dendritic cells transfected with interleukin-12 and tumor-associated antigen messenger RNA induce high avidity cytotoxic T cells. Gene Ther..

[8] Pardi N, Hogan MJ, Porter FW, Weissman D (2018). mRNA vaccines - a new era in vaccinology. Nat Rev Drug Discov..

[9] Sahin U, Karikó K, Türeci Ö (2014). mRNA-based therapeutics--developing a new class of drugs. Nat Rev Drug Discov..

[10] Sahin U, Derhovanessian E, Miller M, Kloke B-P, Simon P, Löwer M (2017). Personalized RNA mutanome vaccines mobilize poly-specific therapeutic immunity against cancer. Nature..

[11] Sahin U, Muik A, Derhovanessian E, Vogler I, Kranz LM, Vormehr M (2020). COVID-19 vaccine BNT162b1 elicits human antibody and TH1 T cell responses. Nature..

[12] Vormehr M, Schrörs B, Boegel S, Löwer M, Türeci Ö, Sahin U (2015). Mutanome engineered RNA immunotherapy: towards patient-centered tumor vaccination. J Immunol Res..

[13] Hobernik D, Bros M. DNA vaccines-how far from clinical use? Int J Mol Sci. 2018;19(11):3605. 10.3390/ijms19113605. 10.3390/ijms19113605 PMC6274812 30445702

[14] Bloom K, van den Berg F, Arbuthnot P. Self-amplifying RNA vaccines for infectious diseases. Gene Ther. 2020:1–13. 10.1038/s41434-020-00204-y. 10.1038/s41434-020-00204-y PMC7580817 33093657

[15] Caruso HG, Torikai H, Zhang L, Maiti S, Dai J, Do K-A (2016). Redirecting T-cell specificity to EGFR using mRNA to self-limit expression of chimeric antigen receptor. J Immunother..

[16] Reinhard K, Rengstl B, Oehm P, Michel K, Billmeier A, Hayduk N (2020). An RNA vaccine drives expansion and efficacy of claudin-CAR-T cells against solid tumors. Science..

[17] Green MR, Sambrook J (2020). In vitro transcription systems. Cold Spring Harb Protoc.

[18] Milligan JF, Groebe DR, Witherell GW, Uhlenbeck OC (1987). Oligoribonucleotide synthesis using T7 RNA polymerase and synthetic DNA templates. Nucl Acids Res..

[19] Paschal BM, McReynolds LA, Noren CJ, Nichols NM. RNA Polymerases. Curr Protoc Mol Biol. 2008;84 Available from: https://onlinelibrary.wiley.com/doi/abs/10.1002/0471142727.mb0308s84. [cited 2020 Dec 23]. 10.1002/0471142727.mb0308s84 18972390

[20] Shatkin AJ, Manley JL. The ends of the affair: capping and polyadenylation. Nat Struct Biol. 2000;7(10):838–42. 10.1038/79583. 10.1038/79583 11017188

[21] Hornung V, Ellegast J, Kim S, Brzózka K, Jung A, Kato H (2006). 5’-Triphosphate RNA is the ligand for RIG-I. Science..

[22] Muttach F, Muthmann N, Rentmeister A (2017). Synthetic mRNA capping. Beilstein J Org Chem..

[23] Stepinski J, Waddell C, Stolarski R, Darzynkiewicz E, Rhoads RE (2001). Synthesis and properties of mRNAs containing the novel “anti-reverse” cap analogs 7-methyl (3’-O-methyl) GpppG and 7-methyl (3’-deoxy)GpppG. RNA..

[24] Schuberth-Wagner C, Ludwig J, Bruder AK, Herzner A-M, Zillinger T, Goldeck M (2015). A conserved histidine in the RNA sensor RIG-I controls immune tolerance to N1-2′O-methylated self RNA. Immunity..

[25] Holtkamp S, Kreiter S, Selmi A, Simon P, Koslowski M, Huber C (2006). Modification of antigen-encoding RNA increases stability, translational efficacy, and T-cell stimulatory capacity of dendritic cells. Blood..

[26] Strzelecka D, Smietanski M, Sikorski PJ, Warminski M, Kowalska J, Jemielity J (2020). Phosphodiester modifications in mRNA poly(A) tail prevent deadenylation without compromising protein expression. RNA..

[27] Sonenberg N, Hinnebusch AG (2009). Regulation of translation initiation in eukaryotes: mechanisms and biological targets. Cell..

[28] Vagner S, Galy B, Pyronnet S (2001). Irresistible IRES: Attracting the translation machinery to internal ribosome entry sites. EMBO Rep..

[29] Kozak M (1989). The scanning model for translation: an update. J Cell Biol..

[30] Jiang Y, Xu X-S, Russell JE (2006). A Nucleolin-binding 3′ untranslated region element stabilizes β-globin mRNA in vivo. MCB..

[31] Kudla G, Lipinski L, Caffin F, Helwak A, Zylicz M (2006). High guanine and cytosine content increases mRNA levels in mammalian cells. Hurst LD, editor. PLoS Biol.

[32] Mauro VP, Chappell SA, Hacker DL (2018). Considerations in the use of codon optimization for recombinant protein expression. Recombinant protein expression in mammalian cells.

[33] Hunt RC, Simhadri VL, Iandoli M, Sauna ZE, Kimchi-Sarfaty C (2014). Exposing synonymous mutations. Trends Genet..

[34] Pardi N, Muramatsu H, Weissman D, Karikó K, Rabinovich PM (2013). In vitro transcription of long rna containing modified nucleosides. Synthetic messenger RNA and cell metabolism modulation.

[35] Baiersdörfer M, Boros G, Muramatsu H, Mahiny A, Vlatkovic I, Sahin U (2019). A facile method for the removal of dsRNA contaminant from in vitro-transcribed mRNA. Mol Ther - Nucleic Acids..

[36] Lutz J, Lazzaro S, Habbeddine M, Schmidt KE, Baumhof P, Mui BL (2017). Unmodified mRNA in LNPs constitutes a competitive technology for prophylactic vaccines. NPJ Vaccines.

[37] Pardi N, Hogan MJ, Weissman D (2020). Recent advances in mRNA vaccine technology. Curr Opin Immunol..

[38] Gehl J (2003). Electroporation: theory and methods, perspectives for drug delivery, gene therapy and research. Acta Physiol Scand..

[39] Kauffman KJ, Webber MJ, Anderson DG (2016). Materials for non-viral intracellular delivery of messenger RNA therapeutics. J Control Release..

[40] Pardi N, Hogan MJ, Naradikian MS, Parkhouse K, Cain DW, Jones L (2018). Nucleoside-modified mRNA vaccines induce potent T follicular helper and germinal center B cell responses. J Exp Med..

[41] Heidenreich R, Jasny E, Kowalczyk A, Lutz J, Probst J, Baumhof P (2015). A novel RNA-based adjuvant combines strong immunostimulatory capacities with a favorable safety profile. Int J Cancer..

[42] Hoerr I, Obst R, Rammensee HG, Jung G (2000). In vivo application of RNA leads to induction of specific cytotoxic T lymphocytes and antibodies. Eur J Immunol..

[43] Qiu P, Ziegelhoffer P, Sun J, Yang NS (1996). Gene gun delivery of mRNA in situ results in efficient transgene expression and genetic immunization. Gene Ther..

[44] Probst J, Weide B, Scheel B, Pichler BJ, Hoerr I, Rammensee H-G (2007). Spontaneous cellular uptake of exogenous messenger RNA in vivo is nucleic acid-specific, saturable and ion dependent. Gene Ther..

[45] Iavarone C, Ramsauer K, Kubarenko AV, Debasitis JC, Leykin I, Weber ANR (2011). A point mutation in the amino terminus of TLR7 abolishes signaling without affecting ligand binding. J Immunol..

[46] Bevan MJ (2006). Cross-priming. Nat Immunol..

[47] Bevan MJ (1976). Cross-priming for a secondary cytotoxic response to minor H antigens with H-2 congenic cells which do not cross-react in the cytotoxic assay. J Exp Med..

[48] Kurts C, Robinson BWS, Knolle PA (2010). Cross-priming in health and disease. Nat Rev Immunol..

[49] Steinman RM, Witmer MD (1978). Lymphoid dendritic cells are potent stimulators of the primary mixed leukocyte reaction in mice. Proc Natl Acad Sci U S A..

[50] Borst J, Ahrends T, Bąbała N, Melief CJM, Kastenmüller W (2018). CD4+ T cell help in cancer immunology and immunotherapy. Nat Rev Immunol..

[51] Smith CM, Wilson NS, Waithman J, Villadangos JA, Carbone FR, Heath WR (2004). Cognate CD4(+) T cell licensing of dendritic cells in CD8(+) T cell immunity. Nat Immunol..

[52] Bretscher P, Cohn M (1970). A theory of self-nonself discrimination. Science..

[53] Matzinger P (1994). Tolerance, danger, and the extended family. Annu Rev Immunol..

[54] Beutler B (2004). Inferences, questions and possibilities in Toll-like receptor signalling. Nature..

[55] Duthie MS, Windish HP, Fox CB, Reed SG (2011). Use of defined TLR ligands as adjuvants within human vaccines. Immunol Rev..

[56] Schreiber G (2017). The molecular basis for differential type I interferon signaling. J Biol Chem..

[57] Honda K, Takaoka A, Taniguchi T (2006). Type I interferon [corrected] gene induction by the interferon regulatory factor family of transcription factors. Immunity..

[58] Chen N, Xia P, Li S, Zhang T, Wang TT, Zhu J (2017). RNA sensors of the innate immune system and their detection of pathogens. IUBMB Life..

[59] Alexopoulou L, Holt AC, Medzhitov R, Flavell RA (2001). Recognition of double-stranded RNA and activation of NF-kappaB by Toll-like receptor 3. Nature..

[60] Heil F, Hemmi H, Hochrein H, Ampenberger F, Kirschning C, Akira S (2004). Species-specific recognition of single-stranded RNA via toll-like receptor 7 and 8. Science..

[61] Iwasaki A, Medzhitov R (2015). Control of adaptive immunity by the innate immune system. Nat Immunol..

[62] Pepini T, Pulichino A-M, Carsillo T, Carlson AL, Sari-Sarraf F, Ramsauer K (2017). Induction of an IFN-mediated antiviral response by a self-amplifying RNA vaccine: implications for vaccine design. J Immunol..

[63] Karikó K, Buckstein M, Ni H, Weissman D (2005). Suppression of RNA recognition by Toll-like receptors: the impact of nucleoside modification and the evolutionary origin of RNA. Immunity..

[64] Bekeredjian-Ding I, Jego G (2009). Toll-like receptors--sentries in the B-cell response. Immunology..

[65] Diebold SS, Kaisho T, Hemmi H, Akira S, Reis e Sousa C (2004). Innate antiviral responses by means of TLR7-mediated recognition of single-stranded RNA. Science.

[66] Scheel B, Aulwurm S, Probst J, Stitz L, Hoerr I, Rammensee H-G (2006). Therapeutic anti-tumor immunity triggered by injections of immunostimulating single-stranded RNA. Eur J Immunol..

[67] Hua Z, Hou B (2013). TLR signaling in B-cell development and activation. Cell Mol Immunol..

[68] Kowalczyk A, Doener F, Zanzinger K, Noth J, Baumhof P, Fotin-Mleczek M (2016). Self-adjuvanted mRNA vaccines induce local innate immune responses that lead to a potent and boostable adaptive immunity. Vaccine..

[69] Edwards DK, Jasny E, Yoon H, Horscroft N, Schanen B, Geter T (2017). Adjuvant effects of a sequence-engineered mRNA vaccine: translational profiling demonstrates similar human and murine innate response. J Transl Med..

[70] Miao L, Li L, Huang Y, Delcassian D, Chahal J, Han J (2019). Delivery of mRNA vaccines with heterocyclic lipids increases anti-tumor efficacy by STING-mediated immune cell activation. Nat Biotechnol..

[71] Ilyas S, Yang JC (2015). Landscape of Tumor Antigens in T Cell Immunotherapy. J Immunol.

[72] Haen SP, Rammensee H-G (2013). The repertoire of human tumor-associated epitopes — identification and selection of antigens and their application in clinical trials. Curr Opin Immunol..

[73] Türeci Ö, Vormehr M, Diken M, Kreiter S, Huber C, Sahin U (2016). Targeting the heterogeneity of cancer with individualized neoepitope vaccines. Clin Cancer Res.

[74] Garraway LA, Lander ES (2013). Lessons from the cancer genome. Cell..

[75] Martincorena I, Campbell PJ (2015). Somatic mutation in cancer and normal cells. Science..

[76] Leclerc M, Mezquita L, Guillebot De Nerville G, Tihy I, Malenica I, Chouaib S, et al. Recent advances in lung cancer immunotherapy: input of T-cell epitopes associated with impaired peptide processing. Front Immunol. 2019;10 Available from: https://www.ncbi.nlm.nih.gov/pmc/articles/PMC6616108/. [cited 2020 Dec 14]. 10.3389/fimmu.2019.01505 PMC6616108 31333652

[77] Fritsch EF, Hacohen N, Wu CJ (2014). Personal neoantigen cancer vaccines: The momentum builds. Oncoimmunology..

[78] Wolchok JD, Chan TA (2014). Cancer: Antitumour immunity gets a boost. Nature..

[79] Schumacher TN, Schreiber RD (2015). Neoantigens in cancer immunotherapy. Science..

[80] Gubin MM, Zhang X, Schuster H, Caron E, Ward JP, Noguchi T (2014). Checkpoint blockade cancer immunotherapy targets tumour-specific mutant antigens. Nature..

[81] Gubin MM, Artyomov MN, Mardis ER, Schreiber RD (2015). Tumor neoantigens: building a framework for personalized cancer immunotherapy. J Clin Invest..

[82] Heine A, Grünebach F, Holderried T, Appel S, Weck MM, Dörfel D (2006). Transfection of dendritic cells with in vitro-transcribed CMV RNA induces polyclonal CD8+- and CD4+-mediated CMV-specific T cell responses. Mol Ther..

[83] Dörfel D, Appel S, Grünebach F, Weck MM, Müller MR, Heine A (2005). Processing and presentation of HLA class I and II epitopes by dendritic cells after transfection with in vitro-transcribed MUC1 RNA. Blood..

[84] Grünebach F, Kayser K, Weck MM, Müller MR, Appel S, Brossart P (2005). Cotransfection of dendritic cells with RNA coding for HER-2/neu and 4-1BBL increases the induction of tumor antigen specific cytotoxic T lymphocytes. Cancer Gene Ther..

[85] Kreiter S, Vormehr M, van de Roemer N, Diken M, Löwer M, Diekmann J (2015). Mutant MHC class II epitopes drive therapeutic immune responses to cancer. Nature..

[86] Scheiblhofer S, Thalhamer J, Weiss R (2018). DNA and mRNA vaccination against allergies. Pediatr Allergy Immunol..

[87] Roesler E, Weiss R, Weinberger EE, Fruehwirth A, Stoecklinger A, Mostböck S (2009). Immunize and disappear-safety-optimized mRNA vaccination with a panel of 29 allergens. J Allergy Clin Immunol.

[88] Havenar-Daughton C, Lee JH, Crotty S (2017). Tfh cells and HIV bnAbs, an immunodominance model of the HIV neutralizing antibody generation problem. Immunol Rev..

[89] Tam HH, Melo MB, Kang M, Pelet JM, Ruda VM, Foley MH (2016). Sustained antigen availability during germinal center initiation enhances antibody responses to vaccination. Proc Natl Acad Sci U S A..

[90] Collin M, Bigley V (2018). Human dendritic cell subsets: an update. Immunology..

[91] Steinman RM (2012). Decisions about dendritic cells: past, present, and future. Annu Rev Immunol..

[92] Banchereau J, Steinman RM (1998). Dendritic cells and the control of immunity. Nature..

[93] Shortman K, Heath WR (2010). The CD8+ dendritic cell subset. Immunol Rev..

[94] Shortman K, Naik SH (2007). Steady-state and inflammatory dendritic-cell development. Nat Rev Immunol..

[95] Hashimoto D, Miller J, Merad M (2011). Dendritic cell and macrophage heterogeneity in vivo. Immunity..

[96] Balan S, Ollion V, Colletti N, Chelbi R, Montanana-Sanchis F, Liu H (2014). Human XCR1+ dendritic cells derived in vitro from CD34+ progenitors closely resemble blood dendritic cells, including their adjuvant responsiveness, contrary to monocyte-derived dendritic cells. J Immunol..

[97] Diken M, Kreiter S, Kloke B, Sahin U. Current developments in actively personalized cancer vaccination with a focus on RNA as the drug format. In: Michielin O, Coukos G, editors. Progress in tumor research. S. Karger AG; 2015. p. 44–54. Available from: https://www.karger.com/Article/FullText/437184. [cited 2020 Dec 22]. 10.1159/000437184 26383626

[98] Diken M, Kreiter S, Selmi A, Britten CM, Huber C, Türeci Ö (2011). Selective uptake of naked vaccine RNA by dendritic cells is driven by macropinocytosis and abrogated upon DC maturation. Gene Ther..

[99] Kreiter S, Selmi A, Diken M, Koslowski M, Britten CM, Huber C (2010). Intranodal vaccination with naked antigen-encoding RNA elicits potent prophylactic and therapeutic antitumoral immunity. Cancer Res..

[100] Zhou WZ, Hoon DS, Huang SK, Fujii S, Hashimoto K, Morishita R (1999). RNA melanoma vaccine: induction of antitumor immunity by human glycoprotein 100 mRNA immunization. Hum Gene Ther..

[101] KleinJan A (2011). The crucial role of dendritic cells in rhinitis. Curr Opin Allergy Clin Immunol..

[102] Takano K-I, Kojima T, Go M, Murata M, Ichimiya S, Himi T (2005). HLA-DR- and CD11c-positive dendritic cells penetrate beyond well-developed epithelial tight junctions in human nasal mucosa of allergic rhinitis. J Histochem Cytochem..

[103] Phua KKL, Staats HF, Leong KW, Nair SK (2014). Intranasal mRNA nanoparticle vaccination induces prophylactic and therapeutic anti-tumor immunity. Sci Rep..

[104] Klechevsky E (2013). Human dendritic cells — stars in the skin. Eur J Immunol..

[105] Clausen BE, Stoitzner P (2015). Functional specialization of skin dendritic cell subsets in regulating T cell responses. Front Immunol..

[106] Weide B, Carralot J-P, Reese A, Scheel B, Eigentler TK, Hoerr I (2008). Results of the first phase I/II clinical vaccination trial with direct injection of mRNA. J Immunother..

[107] Fotin-Mleczek M, Zanzinger K, Heidenreich R, Lorenz C, Thess A, Duchardt KM (2012). Highly potent mRNA based cancer vaccines represent an attractive platform for combination therapies supporting an improved therapeutic effect. J Gene Med..

[108] Hess PR, Boczkowski D, Nair SK, Snyder D, Gilboa E (2006). Vaccination with mRNAs encoding tumor-associated antigens and granulocyte-macrophage colony-stimulating factor efficiently primes CTL responses, but is insufficient to overcome tolerance to a model tumor/self antigen. Cancer Immunol Immunother..

[109] Oberli MA, Reichmuth AM, Dorkin JR, Mitchell MJ, Fenton OS, Jaklenec A (2017). Lipid nanoparticle assisted mRNA delivery for potent cancer immunotherapy. Nano Lett..

[110] Rittig SM, Haentschel M, Weimer KJ, Heine A, Müller MR, Brugger W (2016). Long-term survival correlates with immunological responses in renal cell carcinoma patients treated with mRNA-based immunotherapy. Oncoimmunology..

[111] Rittig SM, Haentschel M, Weimer KJ, Heine A, Muller MR, Brugger W (2011). Intradermal vaccinations with RNA coding for TAA generate CD8+ and CD4+ immune responses and induce clinical benefit in vaccinated patients. Mol Ther..

[112] Liang S-L, Quirk D, Zhou A (2006). RNase L: its biological roles and regulation. IUBMB Life..

[113] Liang F, Ploquin A, Hernández JD, Fausther-Bovendo H, Lindgren G, Stanley D (2015). Dissociation of skeletal muscle for flow cytometric characterization of immune cells in macaques. J Immunol Methods..

[114] Newman JH, Chesson CB, Herzog NL, Bommareddy PK, Aspromonte SM, Pepe R (2020). Intratumoral injection of the seasonal flu shot converts immunologically cold tumors to hot and serves as an immunotherapy for cancer. Proc Natl Acad Sci U S A..

[115] Van der Jeught K, Joe PT, Bialkowski L, Heirman C, Daszkiewicz L, Liechtenstein T (2014). Intratumoral administration of mRNA encoding a fusokine consisting of IFN-β and the ectodomain of the TGF-β receptor II potentiates antitumor immunity. Oncotarget..

[116] Van Lint S, Goyvaerts C, Maenhout S, Goethals L, Disy A, Benteyn D (2012). Preclinical evaluation of TriMix and antigen mRNA-based antitumor therapy. Cancer Res..

[117] Van Lint S, Wilgenhof S, Heirman C, Corthals J, Breckpot K, Bonehill A (2014). Optimized dendritic cell-based immunotherapy for melanoma: the TriMix-formula. Cancer Immunol Immunother..

[118] Guan S, Rosenecker J (2017). Nanotechnologies in delivery of mRNA therapeutics using nonviral vector-based delivery systems. Gene Ther..

[119] Reichmuth AM, Oberli MA, Jaklenec A, Langer R, Blankschtein D (2016). mRNA vaccine delivery using lipid nanoparticles. Ther Deliv..

[120] Midoux P, Pichon C (2015). Lipid-based mRNA vaccine delivery systems. Expert Rev Vaccines..

[121] Pardi N, Tuyishime S, Muramatsu H, Kariko K, Mui BL, Tam YK (2015). Expression kinetics of nucleoside-modified mRNA delivered in lipid nanoparticles to mice by various routes. J Control Release..

[122] Maaske A, Devos FC, Niezold T, Lapuente D, Tannapfel A, Vanoirbeek JA (2016). Mucosal expression of DEC-205 targeted allergen alleviates an asthmatic phenotype in mice. J Control Release..

[123] Niezold T, Storcksdieck Genannt Bonsmann M, Maaske A, Temchura V, Heinecke V, Hannaman D (2015). DNA vaccines encoding DEC205-targeted antigens: immunity or tolerance?. Immunology.

[124] Kranz LM, Diken M, Haas H, Kreiter S, Loquai C, Reuter KC (2016). Systemic RNA delivery to dendritic cells exploits antiviral defence for cancer immunotherapy. Nature..

[125] van Gisbergen KPJM, Sanchez-Hernandez M, Geijtenbeek TBH, van Kooyk Y (2005). Neutrophils mediate immune modulation of dendritic cells through glycosylation-dependent interactions between Mac-1 and DC-SIGN. J Exp Med..

[126] Sarén P, Welgus HG, Kovanen PT (1996). TNF-alpha and IL-1beta selectively induce expression of 92-kDa gelatinase by human macrophages. J Immunol..

[127] Duewell P, Kisser U, Heckelsmiller K, Hoves S, Stoitzner P, Koernig S (2011). ISCOMATRIX adjuvant combines immune activation with antigen delivery to dendritic cells in vivo leading to effective cross-priming of CD8+ T cells. J Immunol..

[128] Karikó K, Muramatsu H, Welsh FA, Ludwig J, Kato H, Akira S (2008). Incorporation of pseudouridine into mRNA yields superior nonimmunogenic vector with increased translational capacity and biological stability. Mol Ther..

[129] Karikó K, Muramatsu H, Ludwig J, Weissman D (2011). Generating the optimal mRNA for therapy: HPLC purification eliminates immune activation and improves translation of nucleoside-modified, protein-encoding mRNA. Nucleic Acids Res..

[130] Summer H, Grämer R, Dröge P. Denaturing urea polyacrylamide gel electrophoresis (Urea PAGE). J Vis Exp. 2009;(32):1485. 10.3791/1485. 10.3791/1485 PMC3329804 19865070

[131] de Haro C, Méndez R, Santoyo J (1996). The eIF-2alpha kinases and the control of protein synthesis. FASEB J..

[132] Rettig L, Haen SP, Bittermann AG, von Boehmer L, Curioni A, Krämer SD (2010). Particle size and activation threshold: a new dimension of danger signaling. Blood..

[133] Van Lint S, Renmans D, Broos K, Dewitte H, Lentacker I, Heirman C (2015). The ReNAissanCe of mRNA-based cancer therapy. Expert Rev Vaccines..

[134] Schnee M, Vogel AB, Voss D, Petsch B, Baumhof P, Kramps T (2016). An mRNA vaccine encoding rabies virus glycoprotein induces protection against lethal infection in mice and correlates of protection in adult and newborn pigs. PLoS Negl Trop Dis..

[135] Kallen K-J, Heidenreich R, Schnee M, Petsch B, Schlake T, Thess A (2013). A novel, disruptive vaccination technology: self-adjuvanted RNActive(®) vaccines. Hum Vaccin Immunother..

[136] Fotin-Mleczek M, Duchardt KM, Lorenz C, Pfeiffer R, Ojkić-Zrna S, Probst J (2011). Messenger RNA-based vaccines with dual activity induce balanced TLR-7 dependent adaptive immune responses and provide antitumor activity. J Immunother..

[137] Petsch B, Schnee M, Vogel AB, Lange E, Hoffmann B, Voss D (2012). Protective efficacy of in vitro synthesized, specific mRNA vaccines against influenza A virus infection. Nat Biotechnol..

[138] Kanasty R, Dorkin JR, Vegas A, Anderson D (2013). Delivery materials for siRNA therapeutics. Nat Mater..

[139] Lonez C, Vandenbranden M, Ruysschaert J-M (2012). Cationic lipids activate intracellular signaling pathways. Adv Drug Deliv Rev..

[140] Bahl K, Senn JJ, Yuzhakov O, Bulychev A, Brito LA, Hassett KJ (2017). Preclinical and clinical demonstration of immunogenicity by mRNA vaccines against H10N8 and H7N9 influenza viruses. Mol Ther..

[141] Alberer M, Gnad-Vogt U, Hong HS, Mehr KT, Backert L, Finak G (2017). Safety and immunogenicity of a mRNA rabies vaccine in healthy adults: an open-label, non-randomised, prospective, first-in-human phase 1 clinical trial. Lancet..

[142] Coulie PG, Van den Eynde BJ, van der Bruggen P, Boon T (2014). Tumour antigens recognized by T lymphocytes: at the core of cancer immunotherapy. Nat Rev Cancer..

[143] Conry RM, LoBuglio AF, Wright M, Sumerel L, Pike MJ, Johanning F (1995). Characterization of a messenger RNA polynucleotide vaccine vector. Cancer Res..

[144] Boczkowski D, Nair SK, Snyder D, Gilboa E (1996). Dendritic cells pulsed with RNA are potent antigen-presenting cells in vitro and in vivo. J Exp Med..

[145] Ashley DM, Faiola B, Nair S, Hale LP, Bigner DD, Gilboa E (1997). Bone marrow-generated dendritic cells pulsed with tumor extracts or tumor RNA induce antitumor immunity against central nervous system tumors. J Exp Med..

[146] Gilboa E, Vieweg J (2004). Cancer immunotherapy with mRNA-transfected dendritic cells. Immunol Rev..

[147] Van Tendeloo V, Snoeck H-W, Lardon F, Vanham GLEE, Nijs G, Lenjou M (1998). Nonviral transfection of distinct types of human dendritic cells: high-efficiency gene transfer by electroporation into hematopoietic progenitor- but not monocyte-derived dendritic cells. Gene Ther..

[148] Van Driessche A, Van de Velde ALR, Nijs G, Braeckman T, Stein B, De Vries JM (2009). Clinical-grade manufacturing of autologous mature mRNA-electroporated dendritic cells and safety testing in acute myeloid leukemia patients in a phase I dose-escalation clinical trial. Cytotherapy..

[149] Kyte JA, Mu L, Aamdal S, Kvalheim G, Dueland S, Hauser M (2006). Phase I/II trial of melanoma therapy with dendritic cells transfected with autologous tumor-mRNA. Cancer Gene Ther..

[150] Aerts-Toegaert C, Heirman C, Tuyaerts S, Corthals J, Aerts JL, Bonehill A (2007). CD83 expression on dendritic cells and T cells: correlation with effective immune responses. Eur J Immunol..

[151] De Keersmaecker B, Heirman C, Corthals J, Empsen C, van Grunsven LA, Allard SD (2011). The combination of 4-1BBL and CD40L strongly enhances the capacity of dendritic cells to stimulate HIV-specific T cell responses. J Leukoc Biol..

[152] Jansen Y, Kruse V, Corthals J, Schats K, van Dam P-J, Seremet T (2020). A randomized controlled phase II clinical trial on mRNA electroporated autologous monocyte-derived dendritic cells (TriMixDC-MEL) as adjuvant treatment for stage III/IV melanoma patients who are disease-free following the resection of macrometastases. Cancer Immunol Immunother..

[153] Bonehill A, Van Nuffel AMT, Corthals J, Tuyaerts S, Heirman C, François V (2009). Single-step antigen loading and activation of dendritic cells by mRNA electroporation for the purpose of therapeutic vaccination in melanoma patients. Clin Cancer Res..

[154] Wilgenhof S, Corthals J, Heirman C, van Baren N, Lucas S, Kvistborg P (2016). Phase II study of autologous monocyte-derived mRNA electroporated dendritic cells (TriMixDC-MEL) plus ipilimumab in patients with pretreated advanced melanoma. J Clin Oncol..

[155] Benteyn D, Heirman C, Bonehill A, Thielemans K, Breckpot K (2015). mRNA-based dendritic cell vaccines. Expert Rev Vaccines..

[156] Van Tendeloo VF, Van de Velde A, Van Driessche A, Cools N, Anguille S, Ladell K (2010). Induction of complete and molecular remissions in acute myeloid leukemia by Wilms’ tumor 1 antigen-targeted dendritic cell vaccination. Proc Natl Acad Sci..

[157] Berneman ZN, Germonpre P, Huizing MT, Van de Velde A, Nijs G, Stein B (2014). Dendritic cell vaccination in malignant pleural mesothelioma: A phase I/II study. JCO.

[158] Anguille S, Van de Velde AL, Smits EL, Van Tendeloo VF, Juliusson G, Cools N (2017). Dendritic cell vaccination as postremission treatment to prevent or delay relapse in acute myeloid leukemia. Blood..

[159] Bigalke I, Fløisand Y, Solum G, Hønnåshagen K, Lundby M, Anderson K (2015). AML patients in minimal residual disease vaccinated with a novel generation of fast dendritic cells expressing WT-1 and PRAME mount specific immune responses that relate to clinical outcome. Blood..

[160] Lichtenegger FS, Deiser K, Rothe M, Schnorfeil FM, Krupka C, Augsberger C (2016). Induction of antigen-specific T-cell responses through dendritic cell vaccination in AML: Results of a phase I/II trial and Ex vivo enhancement by checkpoint blockade. Blood..

[161] Khoury HJ, Collins RH, Blum W, Stiff PS, Elias L, Lebkowski JS (2017). Immune responses and long-term disease recurrence status after telomerase-based dendritic cell immunotherapy in patients with acute myeloid leukemia. Cancer..

[162] Amin A, Dudek AZ, Logan TF, Lance RS, Holzbeierlein JM, Knox JJ, et al. Survival with AGS-003, an autologous dendritic cell–based immunotherapy, in combination with sunitinib in unfavorable risk patients with advanced renal cell carcinoma (RCC): Phase 2 study results. J Immunother Cancer. 2015;3 Available from: https://www.ncbi.nlm.nih.gov/pmc/articles/PMC4404644/. [cited 2020 Dec 23]. 10.1186/s40425-015-0055-3 PMC4404644 25901286

[163] Sebastian M, von Boehmer L, Zippelius A, Mayer F, Reck M, Atanackovic D (2012). Messenger RNA vaccination and B-cell responses in NSCLC patients. JCO.

[164] Kübler H, Scheel B, Gnad-Vogt U, Miller K, Schultze-Seemann W, vom Dorp F, et al. Self-adjuvanted mRNA vaccination in advanced prostate cancer patients: a first-in-man phase I/IIa study. J Immunother Cancer. 2015;3 Available from: https://www.ncbi.nlm.nih.gov/pmc/articles/PMC4468959/. [cited 2020 Dec 23]. 10.1186/s40425-015-0068-y PMC4468959 26082837

[165] Kongsted P, Borch TH, Ellebaek E, Iversen TZ, Andersen R, Met Ö (2017). Dendritic cell vaccination in combination with docetaxel for patients with metastatic castration-resistant prostate cancer: A randomized phase II study. Cytotherapy..

[166] Kyte JA, Aamdal S, Dueland S, Sæbøe-Larsen S, Inderberg EM, Madsbu UE, et al. Immune response and long-term clinical outcome in advanced melanoma patients vaccinated with tumor-mRNA-transfected dendritic cells. Oncoimmunology. 2016;5 Available from: https://www.ncbi.nlm.nih.gov/pmc/articles/PMC5139630/. [cited 2020 Dec 23]. 10.1080/2162402X.2016.1232237 PMC5139630 27999747

[167] Vik-Mo EO, Nyakas M, Mikkelsen BV, Moe MC, Due-Tønnesen P, Suso EMI (2013). Therapeutic vaccination against autologous cancer stem cells with mRNA-transfected dendritic cells in patients with glioblastoma. Cancer Immunol Immunother..

[168] Lesterhuis WJ, De Vries IJM, Schreibelt G, Schuurhuis DH, Aarntzen EH, De Boer A (2010). Immunogenicity of dendritic cells pulsed with CEA peptide or transfected with CEA mRNA for vaccination of colorectal cancer patients. Anticancer Res..

[169] Bol KF, Figdor CG, Aarntzen EH, Welzen ME, van Rossum MM, Blokx WA, et al. Intranodal vaccination with mRNA-optimized dendritic cells in metastatic melanoma patients. Oncoimmunology. 2015;4 Available from: https://www.ncbi.nlm.nih.gov/pmc/articles/PMC4570143/. [cited 2020 Dec 23]. 10.1080/2162402X.2015.1019197 PMC4570143 26405571

[170] Weide B, Pascolo S, Scheel B, Derhovanessian E, Pflugfelder A, Eigentler TK (2009). Direct injection of protamine-protected mRNA: results of a phase 1/2 vaccination trial in metastatic melanoma patients. J Immunother..

[171] Sahin U, Oehm P, Derhovanessian E, Jabulowsky RA, Vormehr M, Gold M (2020). An RNA vaccine drives immunity in checkpoint-inhibitor-treated melanoma. Nature..

[172] Sebastian M, Schröder A, Scheel B, Hong HS, Muth A, von Boehmer L (2019). A phase I/IIa study of the mRNA-based cancer immunotherapy CV9201 in patients with stage IIIB/IV non-small cell lung cancer. Cancer Immunol Immunother..

[173] Papachristofilou A, Hipp MM, Klinkhardt U, Früh M, Sebastian M, Weiss C (2019). Phase Ib evaluation of a self-adjuvanted protamine formulated mRNA-based active cancer immunotherapy, BI1361849 (CV9202), combined with local radiation treatment in patients with stage IV non-small cell lung cancer. J Immunother Cancer..

[174] Romero P, Banchereau J, Bhardwaj N, Cockett M, Disis ML, Dranoff G (2016). The human vaccines project: A roadmap for cancer vaccine development. Sci Transl Med.

[175] Melero I, Gaudernack G, Gerritsen W, Huber C, Parmiani G, Scholl S (2014). Therapeutic vaccines for cancer: an overview of clinical trials. Nat Rev Clin Oncol..

[176] Bordon Y (2020). An RNA vaccine for advanced melanoma. Nat Rev Immunol..

[177] Fridman WH, Zitvogel L, Sautès-Fridman C, Kroemer G (2017). The immune contexture in cancer prognosis and treatment. Nat Rev Clin Oncol..

[178] Bonehill A, Tuyaerts S, Van Nuffel AMT, Heirman C, Bos TJ, Fostier K (2008). Enhancing the T-cell stimulatory capacity of human dendritic cells by co-electroporation with CD40L, CD70 and constitutively active TLR4 encoding mRNA. Mol Ther..

[179] Diken M, Vormehr M, Grunwitz C, Kreiter S, Türeci Ö, Sahin U, Kramps T, Elbers K (2017). Discovery and subtyping of neo-epitope specific t-cell responses for cancer immunotherapy: Addressing the mutanome. RNA vaccines.

[180] Vormehr M, Diken M, Boegel S, Kreiter S, Türeci Ö, Sahin U (2016). Mutanome directed cancer immunotherapy. Curr Opin Immunol..

[181] Castle JC, Kreiter S, Diekmann J, Löwer M, van de Roemer N, de Graaf J (2012). Exploiting the mutanome for tumor vaccination. Cancer Res..

[182] Sahin U, Türeci Ö (2018). Personalized vaccines for cancer immunotherapy. Science..

[183] Carreno BM, Magrini V, Becker-Hapak M, Kaabinejadian S, Hundal J, Petti AA (2015). Cancer immunotherapy. A dendritic cell vaccine increases the breadth and diversity of melanoma neoantigen-specific T cells. Science..

[184] Ott PA, Hu Z, Keskin DB, Shukla SA, Sun J, Bozym DJ (2017). An immunogenic personal neoantigen vaccine for patients with melanoma. Nature..

[185] Frey AB (2015). Suppression of T cell responses in the tumor microenvironment. Vaccine..

[186] Menter T, Tzankov A (2018). Mechanisms of immune evasion and immune modulation by lymphoma cells. Front Oncol..

[187] Bald T, Landsberg J, Lopez-Ramos D, Renn M, Glodde N, Jansen P (2014). Immune cell-poor melanomas benefit from PD-1 blockade after targeted type I IFN activation. Cancer Discov..

[188] Tumeh PC, Harview CL, Yearley JH, Shintaku IP, Taylor EJM, Robert L (2014). PD-1 blockade induces responses by inhibiting adaptive immune resistance. Nature..

[189] Brown SD, Warren RL, Gibb EA, Martin SD, Spinelli JJ, Nelson BH (2014). Neo-antigens predicted by tumor genome meta-analysis correlate with increased patient survival. Genome Res..

[190] Snyder A, Makarov V, Merghoub T, Yuan J, Zaretsky JM, Desrichard A (2014). Genetic basis for clinical response to CTLA-4 blockade in melanoma. N Engl J Med..

[191] Tran E, Turcotte S, Gros A, Robbins PF, Lu Y-C, Dudley ME (2014). Cancer immunotherapy based on mutation-specific CD4+ T cells in a patient with epithelial cancer. Science..

[192] Rizvi NA, Hellmann MD, Snyder A, Kvistborg P, Makarov V, Havel JJ (2015). Cancer immunology. Mutational landscape determines sensitivity to PD-1 blockade in non-small cell lung cancer. Science..

[193] Wang Y, Zhang L, Xu Z, Miao L, Huang L (2018). mRNA vaccine with Antigen-specific checkpoint blockade induces an enhanced immune response against established melanoma. Mol Ther..

[194] Wolf D, Heine A, Brossart P (2014). Implementing combinatorial immunotherapeutic regimens against cancer: The concept of immunological conditioning. OncoImmunology..

[195] Brito LA, Kommareddy S, Maione D, Uematsu Y, Giovani C, Berlanda Scorza F (2015). Self-amplifying mRNA vaccines. Adv Genet..

[196] Chahal JS, Fang T, Woodham AW, Khan OF, Ling J, Anderson DG (2017). An RNA nanoparticle vaccine against Zika virus elicits antibody and CD8+ T cell responses in a mouse model. Sci Rep..

[197] Geall AJ, Verma A, Otten GR, Shaw CA, Hekele A, Banerjee K, et al. Nonviral delivery of self-amplifying RNA vaccines. Proc Natl Acad Sci U S A. 2012;109(36):14604–9. 10.1073/pnas.1209367109. 10.1073/pnas.1209367109 PMC3437863 22908294

[198] Pardi N, Hogan MJ, Pelc RS, Muramatsu H, Andersen H, DeMaso CR (2017). Zika virus protection by a single low-dose nucleoside-modified mRNA vaccination. Nature..

[199] Akinc A, Querbes W, De S, Qin J, Frank-Kamenetsky M, Jayaprakash KN (2010). Targeted delivery of RNAi therapeutics with endogenous and exogenous ligand-based mechanisms. Mol Ther..

[200] Richner JM, Himansu S, Dowd KA, Butler SL, Salazar V, Fox JM (2017). Modified mRNA vaccines protect against Zika virus infection. Cell.

[201] Chahal JS, Khan OF, Cooper CL, McPartlan JS, Tsosie JK, Tilley LD (2016). Dendrimer-RNA nanoparticles generate protective immunity against lethal Ebola, H1N1 influenza, and Toxoplasma gondii challenges with a single dose. Proc Natl Acad Sci U S A..

[202] Ulmer JB, Geall AJ (2016). Recent innovations in mRNA vaccines. Curr Opin Immunol..

[203] Van Gulck E, Vlieghe E, Vekemans M, Van Tendeloo VFI, Van De Velde A, Smits E (2012). mRNA-based dendritic cell vaccination induces potent antiviral T-cell responses in HIV-1-infected patients. AIDS..

[204] Routy J-P, Boulassel M-R, Yassine-Diab B, Nicolette C, Healey D, Jain R (2010). Immunologic activity and safety of autologous HIV RNA-electroporated dendritic cells in HIV-1 infected patients receiving antiretroviral therapy. Clin Immunol..

[205] Allard SD, De Keersmaecker B, de Goede AL, Verschuren EJ, Koetsveld J, Reedijk ML (2012). A phase I/IIa immunotherapy trial of HIV-1-infected patients with Tat, Rev and Nef expressing dendritic cells followed by treatment interruption. Clin Immunol..

[206] Gandhi RT, Kwon DS, Macklin EA, Shopis JR, McLean AP, McBrine N (2016). Immunization of HIV-1-infected persons with autologous dendritic cells transfected with mrna encoding HIV-1 Gag and Nef: Results of a randomized, placebo-controlled clinical trial. J Acquir Immune Defic Syndr..

[207] Jacobson JM, Routy J-P, Welles S, DeBenedette M, Tcherepanova I, Angel JB (2016). Dendritic cell immunotherapy for HIV-1 infection using autologous HIV-1 RNA: A randomized, double-blind, placebo-controlled clinical trial. J Acquir Immune Defic Syndr..

[208] Gay CL, DeBenedette MA, Tcherepanova IY, Gamble A, Lewis WE, Cope AB (2018). Immunogenicity of AGS-004 dendritic cell therapy in patients treated during acute HIV infection. AIDS Res Hum Retroviruses..

[209] Van Craenenbroeck AH, Smits ELJ, Anguille S, Van de Velde A, Stein B, Braeckman T (2015). Induction of cytomegalovirus-specific T cell responses in healthy volunteers and allogeneic stem cell recipients using vaccination with messenger RNA-transfected dendritic cells. Transplantation..

[210] Perri S, Greer CE, Thudium K, Doe B, Legg H, Liu H (2003). An alphavirus replicon particle chimera derived from venezuelan equine encephalitis and sindbis viruses is a potent gene-based vaccine delivery vector. JVI..

[211] Mulligan MJ, Lyke KE, Kitchin N, Absalon J, Gurtman A, Lockhart S (2020). Phase I/II study of COVID-19 RNA vaccine BNT162b1 in adults. Nature..

[212] Polack FP, Thomas SJ, Kitchin N, Absalon J, Gurtman A, Lockhart S, et al. Safety and efficacy of the BNT162b2 mRNA Covid-19 vaccine. N Engl J Med. 2020. 10.1056/NEJMoa2034577 PMC7745181 33301246

[213] Walsh EE, Frenck RW, Falsey AR, Kitchin N, Absalon J, Gurtman A (2020). Safety and immunogenicity of two RNA-based Covid-19 vaccine candidates. N Engl J Med..

[214] Boraschi D, Del Giudice G, Dutel C, Ivanoff B, Rappuoli R, Grubeck-Loebenstein B (2010). Ageing and immunity. Vaccine..

[215] Vogel AB, Kanevsky I, Che Y, Swanson KA, Muik A, Vormehr M, et al. Immunogenic BNT162b vaccines protect rhesus macaques from SARS-CoV-2. Nature. 2021. 10.1038/s41586-021-03275-y 33524990

[216] Nestle FO, Conrad C, Tun-Kyi A, Homey B, Gombert M, Boyman O (2005). Plasmacytoid predendritic cells initiate psoriasis through interferon-alpha production. J Exp Med..

[217] Hwang S-H, Lee H, Yamamoto M, Jones LA, Dayalan J, Hopkins R (2012). B cell TLR7 expression drives anti-RNA autoantibody production and exacerbates disease in systemic lupus erythematosus–prone mice. JI..

[218] Stadler CR, Bähr-Mahmud H, Celik L, Hebich B, Roth AS, Roth RP (2017). Elimination of large tumors in mice by mRNA-encoded bispecific antibodies. Nat Med..

[219] Cully M (2020). Driving CARs to last. Nat Rev Drug Discov.

